# Mechanochemical Synthesis of Advanced Materials for All-Solid-State Battery (ASSB) Applications: A Review

**DOI:** 10.3390/polym17172340

**Published:** 2025-08-28

**Authors:** Zhiming Qiang, Junjun Hu, Beibei Jiang

**Affiliations:** Department of Electrical and Computer Engineering, Kennesaw State University, 840 Polytechnic Lane, Marietta, GA 30060, USA; zqiang1@students.kennesaw.edu (Z.Q.); jhu10@students.kennesaw.edu (J.H.)

**Keywords:** mechanochemical synthesis, solid-state electrolyte, ball milling

## Abstract

Mechanochemical methods have received much attention in the synthesis and design of all-solid-state battery materials in recent years due to their advantages of being green, efficient, easy to operate, and solvent-free. In this review, common mechanochemical methods, including high-energy ball milling, twin-screw extrusion (TSE), and resonant acoustic mixing (RAM), are introduced with the aim of providing a fundamental understanding of the subsequent material design. Subsequently, the discussion focuses on the application of mechanochemical methods in the construction of solid-state electrolytes, anode materials, and cathode materials, especially the research progress of mechanical energy-induced polymerization strategies in building flexible composite electrolytes and enhancing interfacial stability. Through the analysis of representative work, it is demonstrated that mechanochemical methods are gradually evolving from traditional physical processing tools to functional synthesis platforms with chemical reaction capabilities. This review systematically organizes its development and research trends in the field of all-solid-state battery materials and explores potential future breakthrough directions.

## 1. Introduction

The increasing energy demands of modern society coupled with environmental impacts of fossil fuel dependence and the escalating global energy crisis necessitate a rapid shift toward sustainable energy solutions. In this context, rechargeable batteries have emerged as crucial components in enabling energy storage and support decarbonization across various sectors. However, conventional lithium-ion batteries (LIBs), which utilize flammable liquid electrolytes and rely on limited lithium resources, face critical challenges related to safety, cost, and material sustainability [1].

**Figure 1:**
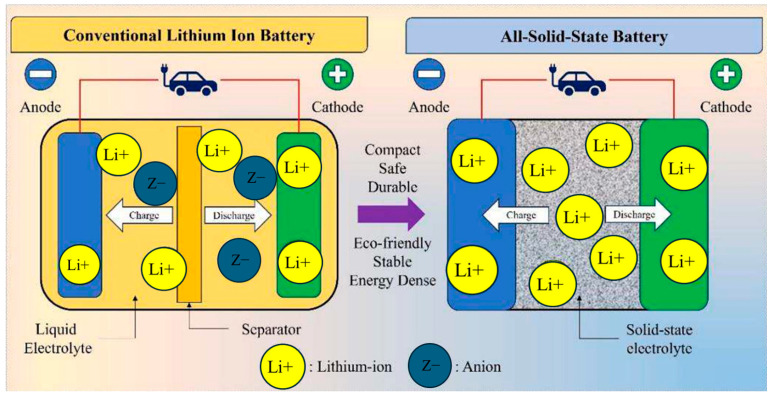
Comparison of conventional and all-solid-state battery. Reprinted with permission from A. Joshi et al. [2], Applied Energy, 386, 125546 (2025). © 2025 Elsevier.

All-solid-state batteries (ASSBs), formed by replacing volatile, flammable liquid electrolytes with chemically stable solid-state electrolytes (SEs), represent the foundation of next-generation batteries due to improved battery safety, eco-friendly, thermal stability, and extended lifespan. As shown in Figure 1, the solid-state electrolyte (SE) serves two roles by conducting Li-ions during repeated charging and discharging while physically separating anodes and cathodes for preventing the short-circuiting risk [2]. Despite their advantages, the commercialization of ASSBs remains limited, largely due to the challenges associated with SEs, including insufficient Li-ion conductivity, limited electrochemical stability, poor compatibility with Li metal, inadequate mechanical strength, and poor interfacial contact with electrodes [3,4,5,6]. Ongoing research aims to overcome these limitations through the development of high-performance SEs, either by innovating new materials chemistries to achieve superionic materials or by employing advanced fabrication methods.

Traditionally, SE materials are synthesized via wet-chemical methods followed by high-temperature sintering or annealing processes. Those wet-chemical methods, such as the sol–gel method [7], co-precipitation method, hydrothermal synthesis [8], and solution combustion synthesis [9], offer precise control over composition, particle morphology, and phase purity. Despite these advantages, these methods face several limitations that hinder their scalability and practical implementation. Key challenges include sluggish Li-ion transport at solid–solid interfaces, often caused by poor interfacial contact, grain boundary resistance, and the formation of space charge layers. Furthermore, the role of point defects and grain boundaries in influencing ion conduction is not yet fully understood, limiting the rational design of SEs with optimized ion transport at the atomic scale [10]. Additionally, the subsequent high-temperature sintering steps typically involve prolonged processing time and substantial energy consumption, making them economically unfavorable and posing further challenges for scalability and interface control [11].

Mechanochemical methods have gained lots of attention for their solvent-free, scalable, and environmentally friendly nature. By harnessing mechanical energy, typically through high-energy ball milling process, these techniques facilitate the formation of amorphous or nanostructured SEs with enhanced ionic transport properties. Recent studies demonstrate that mechanochemistry not only facilitates the formation of novel electrolyte compositions but also improves interfacial contact and conductivity.

The main objective of this review is to provide a comprehensive overview of recent advances in the mechanochemical synthesis of solid-state battery components, including solid electrolytes, cathode materials, and anode materials.

In this review, we strive to provide a comprehensive overview of materials used in ASSBs, organized by their key components, including SEs, anodes, and cathodes. We discuss the desired properties, existing challenges, and both conventional and mechanochemical preparation methods, supported by representative research examples. Furthermore, the fundamental principles and mechanisms of mechanochemical synthesis are introduced, with emphasis on its energy input modes, reaction characteristics, processing conditions, and advantages over conventional thermal or solution-based methods. Subsequently, recent advances in mechanochemistry-assisted synthesis of various components in ASSBs are systematically reviewed, including SEs, cathodes, and anodes. Finally, the effects of mechanochemical conditions on material structure, interface stability, and electrochemical performance are also discussed in detail.

## 2. Mechanochemical Synthesis Method

Mechanochemical synthesis, which is a subfield of the mechanochemistry, induces chemical reactions by applying mechanical energy to the reactants to synthesis materials. Unlike traditional activation methods that rely on thermal, photonic, or electrical energy, mechanochemistry offers a distinct and efficient pathway for driving chemical transformations [12]. Such approach aligns with the principles of green chemistry, promoting solvent-free and environmentally friendly processes. To date, this green approach has demonstrated considerable success. For example, mechanical ball milling has been effectively employed to synthesize metal oxide nanoparticles and polymers under solvent-free conditions [13,14].

### 2.1. Fundamental Principles of Mechanochemistry

The formation and breaking of chemical bonds lie at the core of chemical reactions. For a reaction to take place, the reaction system must overcome an activation energy barrier, which can be supplied by either internal or external energy sources. For example, according to classic Arrhenius equation which revealed the close relationship between temperature and the rate of chemical reactions [15], thermal energy increases the proportion of molecules with sufficient energy to surpass this barrier. Such activation energy barrier represents the minimum energy threshold required to initiate a chemical transformation [12].

**Figure 2:**
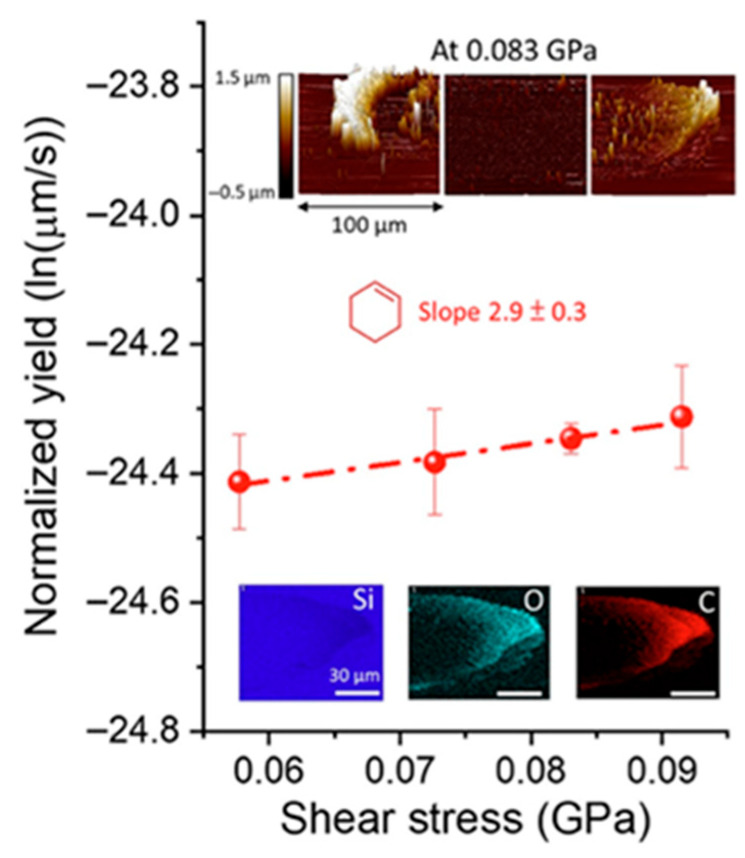
The semi-logarithmic plot showing the normalized yield of tribopolymers as a function of shear stress. Reprinted from Bhuiyan et al. [17], Scientific Reports, 2024, 14, 2992, licensed under CC BY 4.0.

Mechanochemistry offers an alternative pathway to activate chemical reactions by applying mechanical energy directly to the reactants. In processes such as ball milling, the mechanical energy generated by the ball milling machine is transferred to the reactants directly, which can alter chemical bonds or disrupt lattice structures, effectively lowering the activation energy required for the reaction to proceed [16,17]. For example, Fakhrul H. Bhuiyan et al. conducted ball-plane friction experiments to investigate the impact of shear stress on the oligomerization reaction of cyclohexene on a silica surface. As shown in Figure 2, the detected oxygen-containing product exhibits an exponential increase in reaction yield with increasing shear stress, suggesting that shear stress during mechanochemical process could lower reaction barriers and promote chemical transformations [17].

### 2.2. Common Mechanochemistry Equipment, Techniques, and Process Parameters

Traditionally, mechanochemical processes have been predominantly carried out through ball milling, which has played a pivotal role in shaping the field of mechanochemistry. However, mechanochemistry has expanded into a diverse field comprising multiple techniques that deliver mechanical energy in distinct modes for driving chemical reactions [18]. To provide a comprehensive understanding of this field, it is essential to review the major mechanochemical methods and the equipment used to implement them. These techniques can be broadly classified based on their energy input modes and operational configurations. In this section, we present an overview of the most widely adopted mechanochemical approaches, followed by representative examples that illustrate their practical applications in materials synthesis.

#### 2.2.1. Mechanical Ball Milling

Ball milling techniques can be classified based on the mode of movement of the milling media and the mechanism of energy transfer [11]. Through this classification philosophy, ball milling methods are typically categorized into four main types: tumbling ball milling [19], planetary ball milling, where the entire milling jar and the milling media in it rotate around a central “sun wheel” while the jar also rotates on its own axis [18], vibration ball milling [20], and agitator beam milling [21]. Table 1 compares the ball milling techniques, highlighting their advantages, limitations, and trade-offs [11].

**Figure 3:**
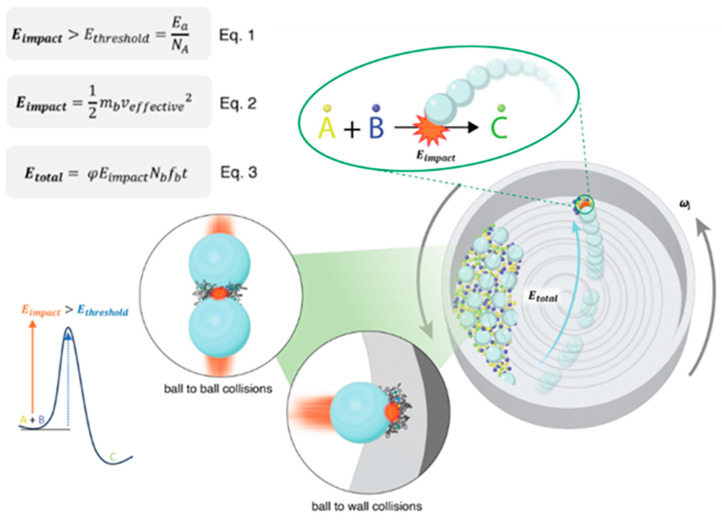
Planetary mill kinematics within the jar. Reproduced from Jafter et al. [18], Angew. Chem. Int. Ed. 2024, with permission from Wiley-VCH.

To understand the mechanistic basis of ball milling and optimize energy input across different setups, it is essential to establish a reliable kinematic model that accurately describes energy transfer. The energy input mode directly influences milling parameters and consequently the reproducibility and scalability of experimental outcomes. To address these challenges, Jafter et al.(2024) [18] proposed a fundamental and comprehensive kinematic energy model that quantitively links milling parameters to total energy input across different ball milling configurations. Their work also introduced a freely accessible online calculator designed to translate parameters between commonly used systems such as planetary and mixer mills, thereby facilitating reproducibility and standardization across laboratories [18]. As illustrated in Figure 3, during planetary ball milling, the milling jar undergoes simultaneous rotation around a central sun wheel and its own axis, resulting in two primary types of collisions: ball-to-ball and ball-to-wall. Both types of collisions generate impact energy that can be delivered to reactant molecules to initiate the chemical reaction. The impact energy (E_impact_), activation energy (E_threshold_), and total energy (E_total_) can be calculated using Equation (1), Equation (2), and Equation (3), respectively, as shown in Figure 3. These equations serve as the fundamental theoretical basis for understanding how mechanical energy is generated, transferred, and utilized in mechanochemical processes. They not only provide a quantitative description of the energy input per collision and the cumulative energy delivered during milling but also establish the critical conditions under which a chemical transformation can be initiated. Below is the explanation of these equations in detail.

(1) $$E_{impact}>E_{treshold}=\frac{E_{a}}{N_{a}}$$

Equation (1) defines that the energy generated from a single ball to molecules collision (E_impact_) which is derived from Equation (2) should be higher than the threshold energy (E_threshold_) required to overcome the activation barrier, calculated as the activation energy (E_a_) divided by Avogadro’s number (N_A_).

(2) $$E_{impact}=\frac{1}{2}m_{b}v_{effective}^{2}$$

Equation (2) is the kinetic energy equation which is used to calculate the impact energy per collision (E_impact_) which depends on the mass of the milling ball (m_b_) and the effective velocity at impact (v_effective_). The effective velocity, which determines the impact energy during mechanochemical collisions, can be derived from the kinetic analysis of a ball’s motion in a planetary mill.

(3) $$E_{total}=\varphi E_{impact}N_{b}f_{b}t$$

Equation (3) estimates the total energy input (E_total_) over a given milling duration t as a function of the impact energy, number of balls (N_b_), collision frequency (f_b_), and empirical filling degree of range (φ); when the space of the jar is completely filled with balls, φ is equal to 0, and when the jar is empty, φ is equal to 1.

This establishes a minimum energy criterion for a reaction to proceed during a single collision. For a mechanochemical reaction to proceed, the impact energy must exceed the activation energy barrier (E_impact_ > E_threshold_). The total mechanical energy delivered throughout the milling process (E_total_) reflects the cumulative energy input available to drive chemical reactions.

In addition to commonly considered milling parameters such as speed, duration, and the material or size of the milling jar and balls, other less frequently reported but critical parameters, such as disk radius (r_p_) and jar dimensions (r_j_), also exhibited strong impact on the process [18].

**Figure 4:**
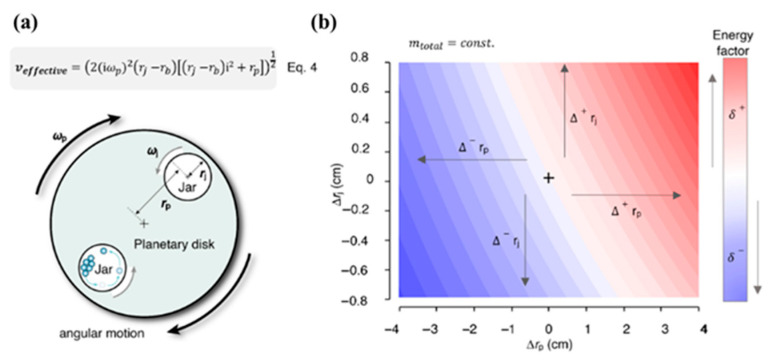
(**a**) Schematics of the ball milling specific parameters and impact energy mapped against these parameters: plate and jar radii in a planetary mill. (**b**) Impact energy distribution as a function of planetary mill geometry, showing variations with respect to jar radius r_j_ and ball position r_p_. Reproduced from Jafter et al. [18], Angew. Chem. Int. Ed. 2024, with permission from Wiley-VCH.

It is worth mentioning that Eq.4 shown in Figure 4a provides a simplified estimation of the effective impact velocity (V_effective_) for a ball inside a planetary ball mill. It is derived from a geometrical and kinematic analysis of the ball’s motion under dual rotation: the spinning of the jar and the rotation of the planetary disk. The equation considers the radial offset between the jar center and the planetary axis (r_j_ − r_p_) and combines this with the angular velocity of the disk (ω_p_) to estimate the trajectory-dependent linear velocity of the ball at the point of impact. While this model does not account for complex collision dynamics or frictional losses, it is highly useful for comparing milling configurations and assessing how system parameters such as jar radius or rotation speed influence energy delivery. As such, it serves as a valuable predictive tool for mechanochemical process optimization.

(4) $$v_{effective}={\left(2{\left(i\omega_{p}\right)}^{2}\left(r_{j}-r_{b}\right)\left[\left(r_{j}-r_{b}\right)i^{2}+r_{p}\right]\right)}^{\frac{1}{2}}$$

As predicted by Equation (4), these geometrical factors directly influence the effective impact velocity (v_effective_), which in turn affects both the impact and total energy transferred to the milling system. In addition, even some minor variations in r_p_ and r_j_ can lead to significant changes in energy input, as demonstrated in Figure 4b. The kinematic energy model mentioned above by Jafter et al. was also validated through two representative mechanochemical experiments: the tert-BuOK-catalyzed amidation of ethyl benzoate with morpholine [22] and a palladium-catalyzed C-C-coupling reaction [13]. These reactions were conducted using various types of ball mills, and in all cases, efficient product formation was observed once the critical energy threshold was exceeded. The consistency between experimental results and theoretical predictions confirms the validity of the proposed model and highlights its potential to enhance reproducibility and guide the optimization of mechanochemical processes.

**Figure 5:**
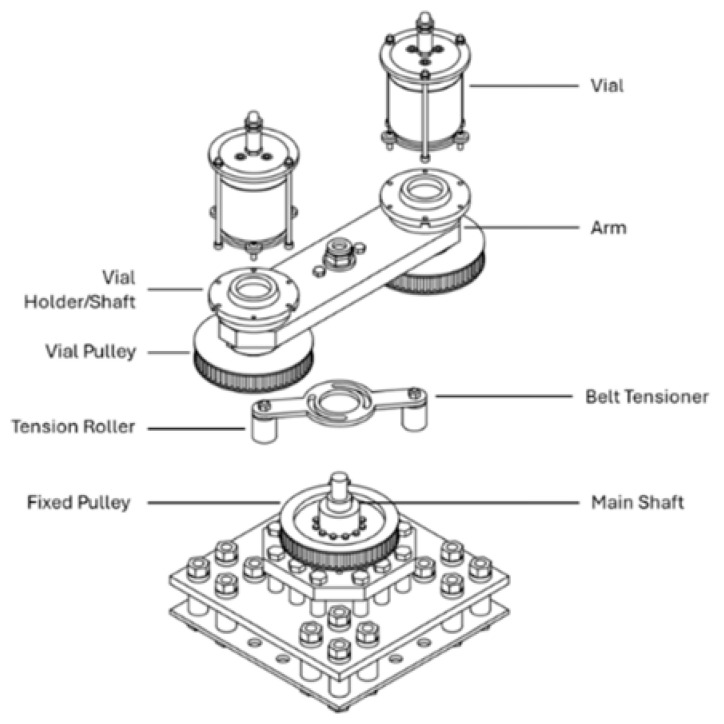
Milling drive system. Reproduced from Yntema et al. [23], RSC Mechano. Chem., 2025, 2, 20–24, with permission from the Royal Society of Chemistry.

Furthermore, apart from traditional ball milling techniques, recent advances in ball milling-based technologies have focused on achieving greater energy efficiency, tunability, and precision. One limitation of traditional planetary ball mills is the fixed ratio between the rotational speeds of the jar and the sun wheel, which restricts control over the energy input. To address this, Yntema et al. reported a new design of planetary ball milling device by applying a modular drive system that allows the user to systematically tune the relative rotation speed between jar and sun wheel by changing the pulleys under the vials, as demonstrated in Figure 5. By enabling manual adjustment of the speed ratio, the newly developed device allows researchers to precisely control the balance between impact energy and milling frequency—two critical parameters that govern mechanochemical reaction efficiency and selectivity. Their findings demonstrate that even subtle changes in milling parameters can substantially affect reaction outcomes, underscoring the complexity of mechanochemical systems. This innovation represents a significant step toward more controlled and reproducible mechanochemical processes [23].

#### 2.2.2. Resonant Acoustic Mixing

**Figure 6:**
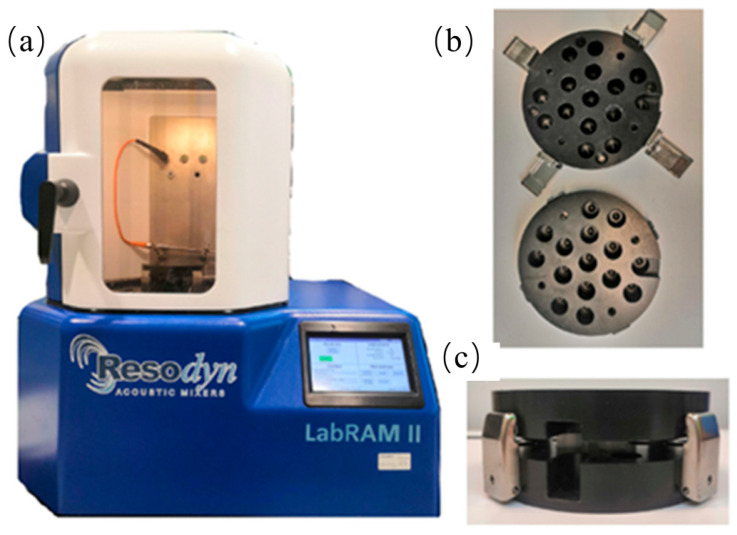
(**a**) LabRAM II resonant acoustic mixing device. (**b**) Top view. (**c**) Side view. Adapted from Gonnet et al. [24], Angew. Chem. Int. Ed., 2022, 61, e202115030, with permission from Wiley.

Resonant acoustic mixing (RAM) is a rapid and energy-efficient mixing technique for inducing material blending and mechanochemical reactions through mechanical agitation, without the need for milling media such as balls, as shown in Figure 6 [24].

By avoiding milling media, it holds great advantages of over conventional ball milling equipment, including avoiding contamination from the milling media, simplifying experimental design, and improved scalability for industrial applications [25]. It enables rapid, uniform, and energy-efficient mixing—even for powders with varying densities—within a closed system that can be easily maintained under inert or controlled atmospheres. RAM is particularly well-suited for sensitive or reactive materials, as it applies high-frequency, low-impact acoustic energy that minimizes thermal degradation.

Understanding the internal dynamics of Resonant Acoustic Mixing (RAM) is essential for optimizing its performance and expanding its application in advanced material processing. A clear understanding of RAM’s internal mechanisms and parameter dependencies is essential for moving beyond a “black box” approach [26]. To address this gap, Sezer et al. conducted the first in-depth study of RAM dynamics by applying positron emission particle tracking (PEPT) with (microcrystalline cellulose) MCC as the testing material. This non-invasive imaging method allowed the researchers to visualize three-dimensional particle motion within the RAM system across various fill levels and vibrational accelerations. Their results revealed robust convective flow patterns and rapid, effective mixing, achieved within one minute across all tested conditions. A pair of symmetric convective circulation structures in the x-z plane and recirculatory motion in the x-y plane were observed for almost all tested combinations of vibration intensity and frequency.

These findings provide unprecedented insights into the physical mechanisms driving RAM, validating its efficiency and offering a foundation for further optimization in applications such as mechanochemistry and solvent-free materials synthesis, and also demonstrate that mixing in RAM follows a regular, predictable macroscopic flow field rather than being random or chaotic. This convective mixing mechanism helps to disperse the material quickly and evenly.

#### 2.2.3. Twin-Crew Extrusion

**Figure 7:**
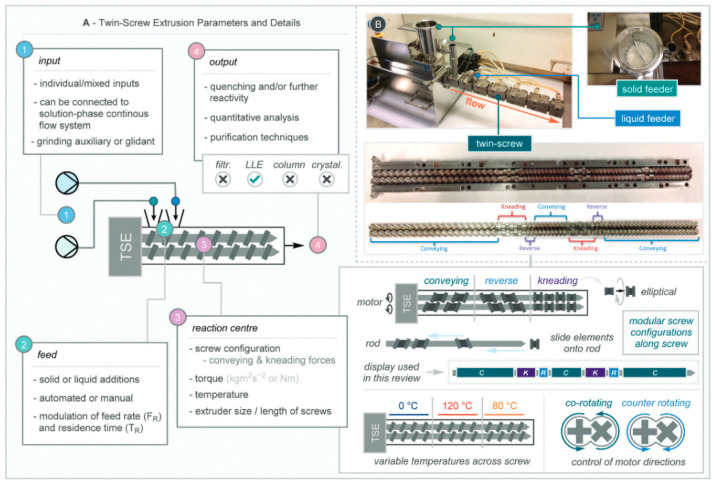
Structure of TSE. Reprinted from Bolt et al. [27]. (**A**) Schematic illustration of the main parameters and details of the twin-screw extrusion process. (**B**) Photograph of the twin-screw extrusion setup, showing the solid and liquid feeders and the twin-screw barrel. Chem. Soc. Rev., 2022, 51, 4243–4260, with permission from the Royal Society of Chemistry.

Traditional ball milling is often limited by low scalability due to its batch processing nature. Reactive extrusion, particularly twin-screw extrusion (TSE), offers a promising solution by enabling continuous “flow-mode” mechanochemical synthesis. TSE employs two co-rotating or counter-rotating screws to continuously mix and process reactants within a closed chamber. One generally accepted structure mode of TSE was proposed by Bolt et al., in which they divided this system into four key sections, including input, feed, reaction center, and output [27]. As shown in Figure 7A, the input section describes where raw materials, possibly pre-mixed by ball milling, along with solvents or additives, are introduced. The feed section describes the addition to the feeder with manual or automated input. The reaction center stands for the closed chamber housing the two screws powered by a motor for controlling the screw speed and the torque. The reaction center can be modularly configured along the screw shaft into conveying, reverse, and kneading zones, each with independently controlled temperatures, and the screws can be set to rotate either co-directionally or counter-directionally, as shown in Figure 7B. Finally, the output section is where the processed product, typically in powder or paste form, is discharged for further analysis. This technique marks meaningful milestone in mechanochemical synthesis by transforming from old fashion “batch” to continuous “flow” mode which can scale up the production output. Plus, reactive extrusion has been acknowledged by IUPAC as one of the ten chemical innovations which can change the world [28].

### 2.3. Mechanochemistry 2.0: Hybrid Energy-Assisted Approaches

The integration of mechanical force with additional energy inputs—such as thermal, photonic, ultrasonic, or electrical stimulation—has attracted increasing attention, enabling chemical transformations that are not feasible with conventional mechanochemical methods alone. These hybrid approaches, often referred to as thermal-, photo-, sono-, or electro-mechanochemistry, have led to the development of specialized experimental setups tailored to harness these combined energy modes [29]. Based on the additional energy input source, these techniques can be categorized into thermal/photo/sono/electro-mechanochemistry [29]. However, their broader application remains limited by technical challenges such as equipment complexity, poor standardization, and difficulties in controlling and integrating multiple energy inputs. For example, one practical challenge for thermo-mechanochemistry is the rapid motion of the milling vessel and the intense impact forces generated by the grinding media during operation. It would be desirable for materials used in reactors to have strong durability against mechanical wear. Another challenge in non-direct methods like photo-mechanochemistry is the opacity of conventional milling vessels, which hinder light transmission. However, this issue can be potentially resolved by using modern transparent materials such as advanced polymers or hardened glass for the vessel and impeller [29]. These challenges have so far limited the widespread application of hybrid mechanochemical methods, but they also present important directions for future research and reactor development.

## 3. Applications of Mechanochemically Synthesized Materials

Mechanochemistry has emerged as a versatile and sustainable approach for material synthesis across a range of fields. In organic synthesis, it offers environmentally friendly and solvent-free routes with enhanced efficiency. For example, Kubota et al. developed a generalized solid-state Suzuki–Miyaura coupling strategy based on the liquid-assisted ball milling (LAG) method. In such mechanochemical method, the solid aryl halides and arylboronic acids are co-milled to achieve carbon–carbon bond formation under solvent-free conditions, while traditionally it is carried out in solution-phase synthesis [30].

Mechanochemical methods are also widely applied in the synthesis of metal–organic frameworks (MOFs), offering advantages such as reduced solvent usage, faster reaction times, and minimal waste generation [31]. In Milner et al.’s work, the authors demonstrated the solid-state synthesis of MOF-74 isomer Mg_2_(m-dobdc) and the expanded framework Mg_2_(dobpdc) for the first time by using ball milling-assisted synthesis employing tungsten carbide vessels and milling media [32]. Remarkably, the materials synthesized at 2 mmol and 8 mmol scales exhibited Langmuir surface areas of 1852 ± 53 m^2^/g and 1992 ± 49 m^2^/g, respectively, which is comparable to those achieved via conventional solvothermal routes (1914 m^2^/g) [27]. Beyond conventional MOF-74 analogues, mechanochemical strategies have also been employed to access more complex framework systems. For example, Karadeniz et al. reported the selective synthesis of either cubic MOF-525 or hexagonal PCN-223 via ball milling by tuning the zirconium precursor and choice of liquid additive. They revealed that MOF-525 can be converted to PCN-223 through milling, which can be attributed to plastic deformations and defect formation during the nucleation of MOF-525 driven by mechanical force [33]. All these examples demonstrated the ability of mechanochemistry to drive solid-state reactions or phase transitions [33].

Mechanochemical synthesis has also emerged as a promising fabrication approach for the synthesis of energy storage materials, particularly in the field of solid-state batteries. For example, sulfide-based solid electrolytes such as Li_3_PS_4_ [34] and Li_7_P_3_S_11_ [35,36] have been synthesized through high-energy ball milling. In addition, cathode materials such as Na_3_(VOPO_4_)_2_F [37] and various polyanionic compounds have been prepared mechanochemically. A more detailed discussion of mechanochemistry-assisted synthesis strategies for solid-state battery components is provided in Section 4.

## 4. Mechanochemical Synthesis for All-Solid-State Battery Materials

In ASSBs, the primary battery material components include solid-state electrolytes (SEs), anodes, and cathodes. Each component plays a critical role in determining the overall performance of the cell and presents unique chemical, physical, and engineering challenges. A comprehensive understanding of the synthesis and processing methods for these materials is essential, as fabrication conditions significantly influence their structural, electrochemical, and interfacial properties. In recent years, mechanochemical synthesis has emerged as a promising strategy for the preparation of ASSB materials, offering environmentally benign, cost-effective, and potentially scalable alternatives to conventional solution- or thermal-based methods. This approach offers a more environmentally friendly, cost-effective, and potentially scalable alternative to conventional synthesis methods. The following sections provide a comprehensive overview of recent advancements in the mechanochemical synthesis of key battery components, including solid-state electrolytes, anodes, and cathodes.

### 4.1. Solid-State Electrolytes (SEs)

**Figure 8:**
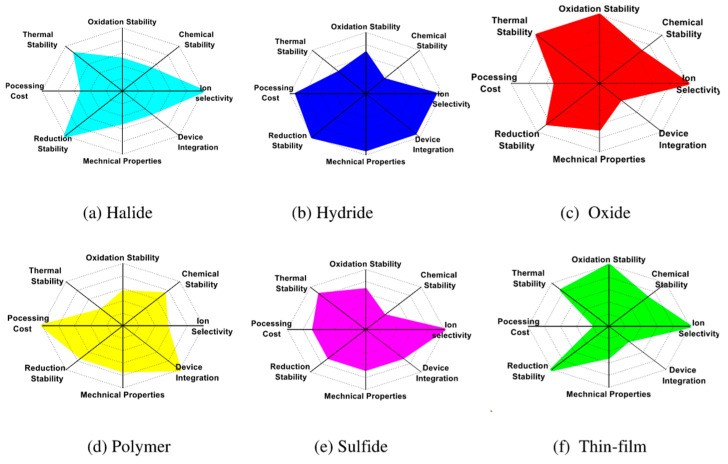
Radar chart for trade-offs in different properties for SSEs. Reproduced with permission from A. Joshi et al. [2], Applied Energy, 386, 125546 (2025). © 2025 Elsevier.

SEs serve as the ion-conducting medium in ASSBs, enabling the transport of Li-ions between electrodes while blocking electron flow to ensure safe and efficient operation. The ideal SEs should exhibit high ionic conductivity, a broad electrochemical stability window, strong chemical and electrochemical compatibility with electrode materials and desirable mechanical strength, flexibility, and thermal stability [38]. Common types of SEs used in ASSBs include oxide-based garnet-type (e.g., Li_7_La_3_Zr_2_O_12_ (LLZO)) [39] and NASICON-type (Li_1_._3_Al_0_._3_Ti_1_._7_(PO_4_)_3_ (LATP)), sulfide-based (e.g., Li_10_GeP_2_S_12_ (LGPS), Li_6_PS_5_Cl) [40], halide-based electrolytes (e.g., Li_3_YCl_6_, Li_3_InCl_6_) [41], and polymer electrolytes based on matrices like poly(ethylene oxide) (PEO), poly(vinylidene fluoride) (PVDF), poly(vinylidene fluoride-co-hexafluoropropylene) (PVDF–HFP), fluorinated polyacrylate copolymers with fluorinated side chains [42,43,44], and ceramic-in-polymer composite electrolytes [45]. Each class of SE exhibits a unique combination of ionic conductivity, chemical stability, mechanical robustness, and compatibility with electrodes, as shown in Figure 8 [2].

Beyond classifying solid-state electrolytes by type, it is equally important to consider how rational design strategies at the material and interface levels can be employed to optimize their performance. For instance, Angulakshmi et al. demonstrated a rational strategy by incorporating MgAl_2_SiO_6_ ceramic filler into a PEO-based polymer matrix, which led to substantial improvements in ionic conductivity, thermal stability, and interfacial compatibility with lithium metal. The resulting all-solid-state Li/CPE/LiFePO_4_ cell delivered a stable capacity of 115 mAh g^−1^ at 1 C and 70 °C, showcasing how material-level engineering contributes directly to electrochemical performance [46]. On the other hand, rational design principles are equally essential at the electrode level. Sathya et al. investigated the effect of different binder formulations (PAA, CMC, and their blends) on the cycling stability of SiO_x_-Si-C composite anodes for Li-S batteries [47]. Their findings revealed that PAA, due to its dense carboxylic functionality and strong hydrogen bonding with SiO_x_ particles, ensured better adhesion, reduced delamination, and higher Coulombic efficiency during long-term cycling. This illustrates how careful molecular design of the binder can address mechanical degradation and SEI instability issues in high-capacity electrodes. These studies exemplify how rational design at both the electrolyte and electrode levels can be synergistically leveraged to overcome key bottlenecks in solid-state battery development.

#### 4.1.1. Challenges Faced by Traditional Synthesis Methods

Conventional synthesis approach includes high-temperature solid-state reactions, as well as solvent-based methods such as sol–gel techniques, and co-precipitation. These traditional synthesis methods often have different limitations. Conventional high-temperature solid-phase synthesis methods often require substantial thermal energy to disrupt chemical bonds and enable atomic rearrangement. These processes are typically energy-intensive, time-consuming, and challenging to scale up for large-scale manufacturing [48]. In one example by Zachary Warren et al., they synthesized Li_6_PS_5_Cl by a solution precipitation method followed by high-temperature open-vessel sintering, achieving an ionic conductivity of 2.99 mS/cm at 550 °C [49]. In contrast, the solvent-based fabrication method offers greater scalability for electrolyte fabrication. However, these approaches often raise safety and environmental concerns due to the use of flammable or toxic solvents such as tetrahydrofuran (THF) and ethanol (EtOH). In some cases, the solvents even react with precursors, leading to the formation of unwanted by-products and compromising material purity. So Yubuchi et al. successfully synthesized Li_6_PS_5_Br solid electrolytes with high ionic conductivity (3.1 mS/cm) using THF and the ethanol solvent method, and they stated that P_2_S_5_ is compromised by EtOH in their experiments [50]. These limitations have prompted growing interest in alternative approaches, particularly mechanochemical synthesis methods such as mechanical mixing and high-energy ball milling. These techniques offer several advantages, including solvent-free processing, reduced energy consumption, and enhanced scalability, making them promising candidates for sustainable and large-scale production of battery materials.

#### 4.1.2. Mechanochemical Synthesis of Halide SEs

Halide SEs, such as Li_3_YCl_6_ or Li_2_ZrCl_6_, have attracted significant attention due to their high electrochemical stability at elevated voltages, soft mechanical properties that allow cold pressing into dense pellets, and favorable ionic conductivity [51]. The mechanochemical method offers the potential to lower energy and time consumption in contrast to traditional high temperature synthesis methods. This approach is particularly beneficial for materials that are thermally sensitive or prone to phase transitions. For example, Li_2_ZnCl_4_ is a typical halide-based SE with two crystalline phases: spinel and olivine type. These two phases undergo a reversible phase transition in the 370 to 475 K range [52]. Notably, Li_2_ZnCl_4_ can be synthesized by mechanical ball milling under milder conditions, avoiding high-temperature processing while enabling phase-selective control [53]. This demonstrates the unique capability of mechanochemistry to induce solid-state transformations and tailor crystal structures without thermal input.

**Figure 9:**
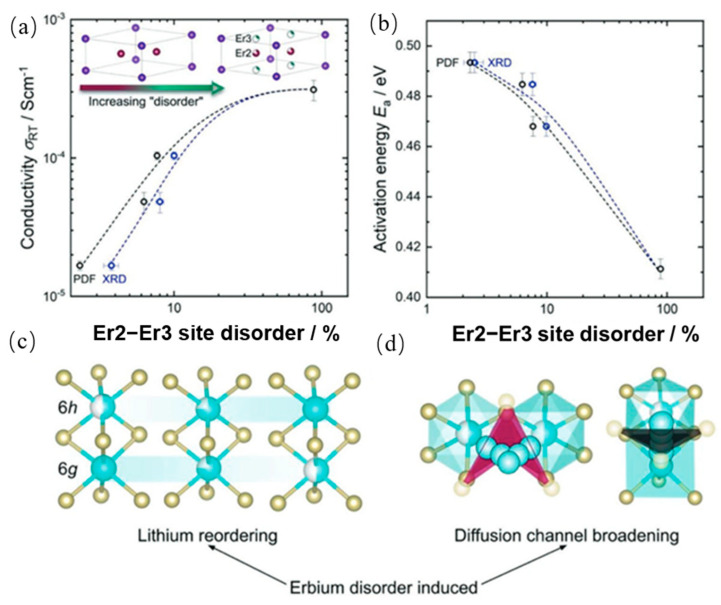
Correlation between cation site disorder (Er2–Er3) and the Li^+^ ionic conductivity (**a**) and activation energy (**b**) in Li_3_ErCl_6_ synthesized via different methods. Schematic illustrations (**c**,**d**) show how increased Er disorder may promote Li sublattice rearrangement and facilitate lithium-ion diffusion. Reproduced with permission from Schlem, R. et al. [55]. Adv. Energy Mater., 2020, 10, 1903719. © Wiley-VCH.

One commonly investigated lithium-conducting halide is Li_3_MX_6_ (M = Y, Sc, Er, Al, Ga, In; X = Cl, Br) [54]. In a study by Roman Schlem et al., the impact of mechanochemical synthesis on the structural and electrochemical properties of Li_3_MCl_6_ (M = Y, Er) was investigated [55]. They discovered that by affecting the local structure and cationic ordering, synthesis methods can influence the transport of lithium ions in the halides. Under ball milling process, Er2 and Er3 site disorder strongly correlates with both enhanced ionic conductivity and reduced activation energy, as shown in Figure 9 [55]. Compared to ampoule synthesis, which yielded a Li-ion conductivity of 1.7 × 10^−5^ S·cm^−1^, the ball milled samples exhibited a substantially higher Li-ion conductivity of 3.1 × 10^−4^ S·cm^−1^. This work highlights that mechanochemical synthesis is not merely a fabrication method but also a powerful tool for structural engineering and performance optimization in halide solid electrolytes.

Mechanochemical synthesis has proven effective not only for rare-earth halide electrolytes like Li_3_YCl_6_, but also for low-cost, rare earth substitution systems. Recently, Li_2_ZrCl_6_ has attracted a lot of attention as a chloride SE composed of the abundant element Zr. Luo et al. [51] systematically investigated the effects of ball milling and mild heat treatment on its structure and properties, finding that a network-like microstructure constructed by nanorods and nanofilaments can be formed after mild heating (100 °C), which can significantly enhance the Li-ion conductivity to 4.46 × 10^−4^ S/cm. Kwak et al. [56] further constructed Li_2_._25_Zr_0_._75_Fe_0_._25_Cl_6_ by Fe^3+^ heterovalent doping via the mechanochemical method, achieving a high conductivity near 1.0 mS/cm and revealing the potential role of Fe-Cl covalency enhancement in lowering the migration barrier. A key significance of this study lies in its successful development of high-performance halide solid electrolytes without relying on rare-earth elements. While most previously reported halide electrolytes—such as Li_3_YCl_6_ or Li_3_InCl_6_—depend on expensive and scarce metals like Y, Er, or In, the authors demonstrate that Li_2_ZrCl_6_ and its Fe^3+^-substituted variant, synthesized via a mechanochemical route, can achieve comparable ionic conductivities (~1 mS cm^−1^) using only earth-abundant and low-cost elements such as Zr and Fe. This not only lowers material costs and enhances scalability but also broadens the practical applicability of halide electrolytes for next-generation all-solid-state batteries.

**Figure 10:**
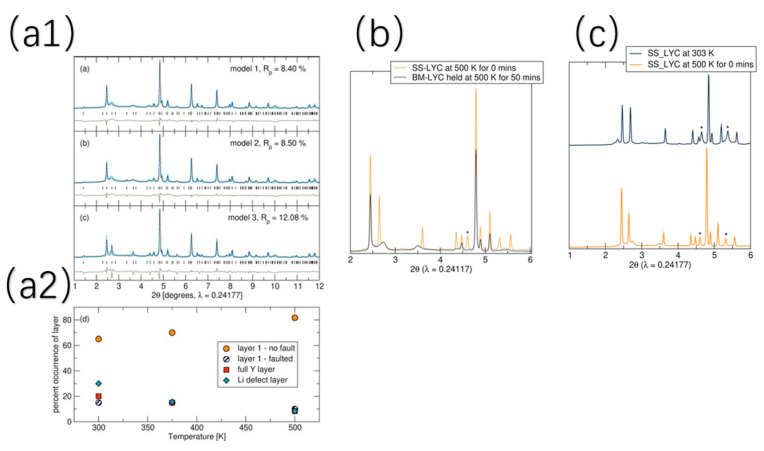
(**a1**) XRD result comparison of BM-LYC, (**a2**) Percentage occurrence of different layers as a function of temperature, (**b**) Diffraction pattern which shows the impurity of SS-LYC, and (**c**) Difference of diffraction pattern of different heating condition of SS-LYC. Adapted with permission from Sebti et al. [57], “Stacking Faults Assist Lithium-Ion Conduction in a Halide-Based Superionic Conductor” Journal of the American Chemical Society, 2022, 144, 5795–5811. Copyright © 2022 American Chemical Society.

A key advantage of mechanochemical synthesis over traditional methods lies in its ability to produce nonequilibrium compounds and introduce structural disorder—such as stacking faults—that significantly enhance lithium-ion conductivity. This is discussed in further detail below. By introducing defect-rich phases, this method creates low-energy migration pathways, making it a powerful approach for optimizing the performance of solid-state electrolytes [57]. For example, Sebti et al. [57] conducted a pivotal study on the Li_3_YCl_6_. They prepared Li_3_YCl_6_ (LYC) using both mechanochemical ball milling (BM-LYC) and traditional solid-state synthesis (SS-LYC) for comparative analysis. They utilized synchrotron X-ray diffraction to characterize Li-Y-Cl superionic conductors, revealing distinct structural differences between mechanochemically synthesized (BM-LYC) and solid-state synthesized (SS-LYC) samples. The XRD patterns of BM-LYC at 303 K (Figure 10(a1,a2)) exhibit peak broadening and asymmetry, indicative of stacking faults (Y and Y/L layers) confirmed by FAULTS refinement, which signify a defect-rich, nonequilibrium structure. In contrast, SS-LYC patterns at 500 K (Figure 10b,c) show multiphase characteristics with lower phase purity, limiting structural refinement.

**Figure 11:**
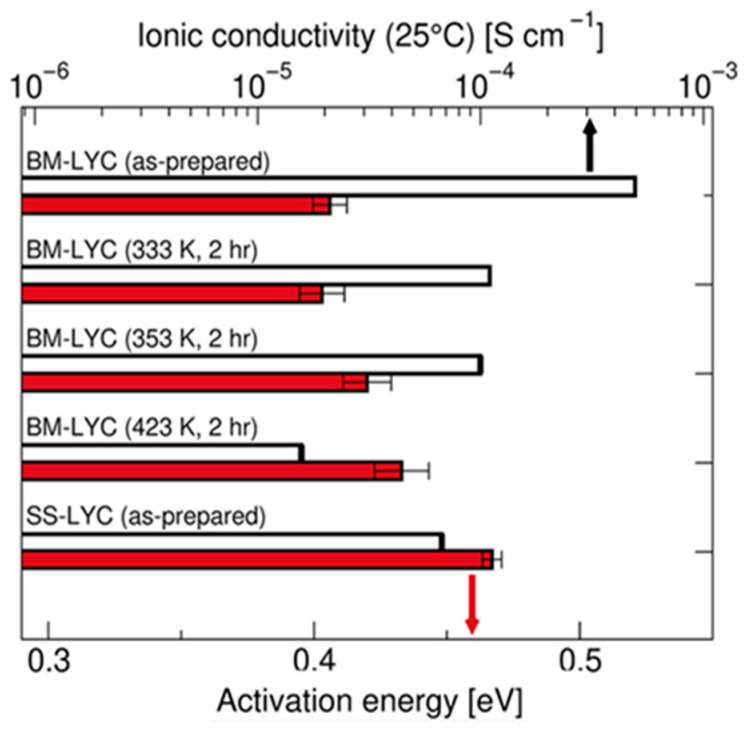
Ionic conductivity and activation energy results of BM-LYC and SS-LYC under room temperature. Adapted with permission from Sebti et al. [57], The black arrow highlights the BM-LYC (as-prepared) sample, which exhibits the lowest activation energy and highest conductivity, whereas the red arrow indicates the SS-LYC (as-prepared) sample, characterized by higher activation energy and lower conductivity. “Stacking Faults Assist Lithium-Ion Conduction in a Halide-Based Superionic Conductor” Journal of the American Chemical Society, 2022, 144, 5795–5811. Copyright © 2022 American Chemical Society.

Figure 11 further proves that mechanochemically synthesized Li_3_YCl_6_ (BM-LYC) exhibits significantly higher ionic conductivity and lower activation energy compared to the solid-state synthesized sample (SS-LYC). This advantage arises from the high concentration of stacking faults and Li-rich defects introduced by ball milling, which create additional Li^+^ conduction pathways and reduce migration barriers. Upon mild heat treatment, these beneficial defects diminish, leading to decreased conductivity.

These results show that mechanochemical methods can effectively tune both the structure and ion transport properties in low-cost halide electrolytes.

#### 4.1.3. Mechanochemical Synthesis of Oxide SEs

Oxide SEs have been widely investigated due to their high energy density, superior mechanical properties, and high stability under high voltages, which can be attributed to their high bandgap [40]. Representative oxide SEs include Garnet type (e.g., Li_7_La_3_Zr_2_O_12_) [58,59], NASICON type (e.g., Li_1_._5_Al_0_._5_Ti_1_._5_(PO_4_)_3_ and Li_1_._5_Al_0_._5_Ge_1_._5_(PO_4_)_3_) [60,61], LISICON type (e.g., Li_4_GeO_4_-Li_3_PO_4_) [62], and Perovskite type (e.g., Li_0_._33_La_0_._56_TiO_3_) [63].

Oxide-based SEs are typically prepared using high-temperature processes such as sintering to achieve the desired phase transitions; however, these methods can cause issues such as mechanical deformation, stoichiometric imbalance, and phase instability [6],which reduce ionic conductivity.

**Figure 12:**
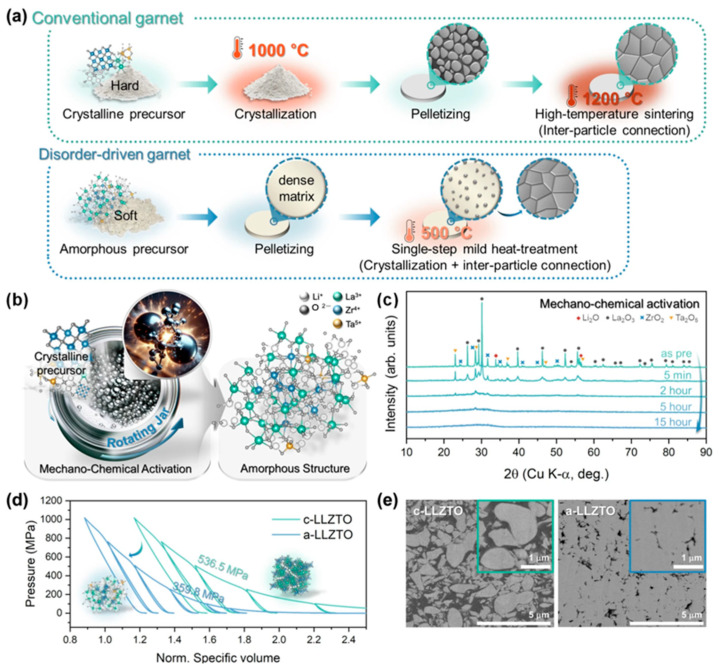
Synthesis and characterization of amorphous LLZTO. (**a**) Preparation schematics comparing the synthesis routes of conventional garnet-type solid electrolytes (top) and disordered garnet (D-garnet, bottom). (**b**) Illustration of the mechanochemical synthesis process used to obtain amorphous LLZTO (a-LLZTO). (**c**) XRD patterns showing the evolution of precursor crystallinity during high-energy ball milling over varying durations. (**d**) Comparison of the powder compaction behavior between crystalline LLZTO (c-LLZTO) and amorphous LLZTO. (**e**) SEM images revealing microstructural differences between c-LLZTO (**left**) and a-LLZTO (**right**). Reproduced from Kwon et al. [64], Nature Communications, 2025, 16, 3256, with permission from Springer Nature.

To address these challenges, mechanochemical methods have emerged as a promising alternative synthetic strategy. These approaches utilize mechanical energy—typically through techniques such as ball milling, grinding, or extrusion—to drive chemical reactions and induce phase transitions or defect formation. Unlike conventional methods that rely on high-temperature treatments, mechanochemical processes can introduce structural disorder or amorphization under ambient conditions, often in the absence of solvents. Such mechanochemical features not only enhance reaction efficiency and energy savings but also broaden the scope for materials design and functional tuning. For example, Kwon et al. introduced a disorder-driven approach to synthesize garnet-type SEs using mechanochemical activation and compared it with conventional methods [64]. As shown in Figure 12c, mechanochemical activation was utilized as a strategy to amorphized Li_6_._5_La_3_Zr_1_._5_Ta_0_._5_O_12_ (LLZTO). After 15 h of milling the precursor, the crystal structure was fully amorphized. Upon subsequent heating, the amorphous LLZTO (a-LLZTO) exhibited an exothermic crystallization event near 400 °C, corresponding to the formation of the cubic garnet phase. Such reduced crystallization temperature can be attributed to the elevated energy state and structural disorder introduced by the mechanochemical process, which lowers the activation energy for phase transformation. As a result, the disorder-driven garnet (D-garnet) obtained at 600 °C achieved an Li-ion conductivity of 3.5 × 10^−4^ S/cm, which is three orders of magnitude higher than that of conventionally sintered samples, with a reduced activation energy of 0.368 eV [64].

**Figure 13:**
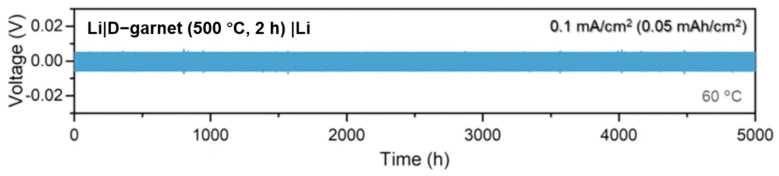
Stripping and plating behavior of symmetric cell of D-garnet electrolyte. Adapted from Kwon et al. [64], Nature Communications, 2025, 16, 3256, with permission from Springer Nature.

In Kwon’s study, symmetric cell testing using a Li|D-garnet|Li configuration was conducted to evaluate the interfacial stability and electrochemical robustness of the disorder-driven garnet electrolyte (Figure 13). Remarkably, the cell demonstrated highly reversible lithium plating and stripping behavior over 5000 cycles at a current density of 0.1 mA/cm^2^ and 60 °C, with a stable overpotential of approximately 5 mV throughout the test. This outstanding cycling stability—achieved without any external pressure during operation—highlights the strong mechanical integrity and excellent interfacial compatibility of the D-garnet with lithium metal.

**Figure 14:**
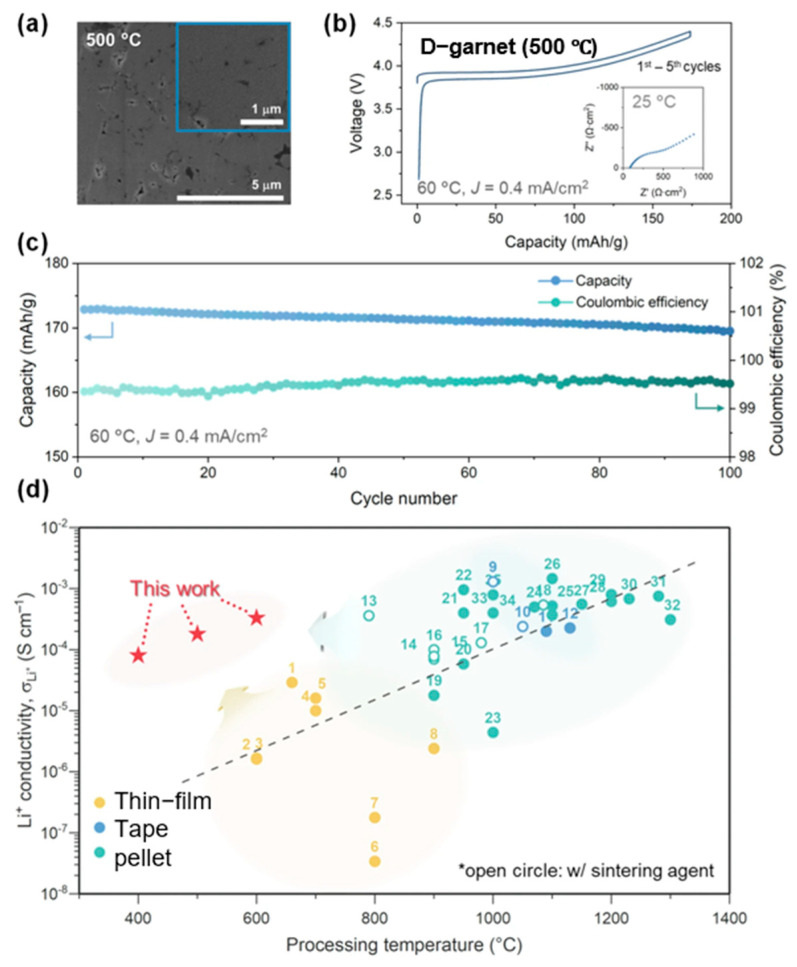
Electrochemical data of D-garnet SE. (**a**) SEM image of D-garnet solid electrolyte. (**b**) Electrochemical voltage profiles of the first five cycles. (**c**) Cycling stability of a hybrid Li-metal cell using D-garnet electrolyte and LCO cathode at 60 °C, operated at 0.4 mA/cm^2^ (~1.15 C) after an initial formation cycle at 0.1 mA/cm^2^ (~0.29 C). (**d**) Comparative plot of ionic conductivity versus processing temperature for various garment-based solid electrolytes. reproduced from Kwon et al. [64], Nature Communications, 2025, 16, 3256, with permission from Springer Nature.

From an application perspective, the electrochemical results presented in Figure 14 [64] clearly highlight the practical advantages of the disorder-driven garnet (D-garnet) solid electrolyte. The SEM image in Figure 14a shows a dense microstructure achieved at just 500 °C, confirming the advantage of the low-temperature mechanochemical route. Electrochemical profiles in Figure 14b,c demonstrate high initial capacity (173.9 mAh/g), excellent Coulombic efficiency (~99.5–100%), and stable cycling over 100 cycles. Importantly, the conductivity–temperature plot in Figure 14d shows that this method achieves high ionic conductivity (10^−4^ to 10^−3^ S/cm) at much lower processing temperatures, overcoming the typical trade-off between conductivity and sintering temperature.

The electrochemical results in Kwon’s study underscore the unique advantages of the mechanochemical approach in solid-state electrolyte design. By leveraging structural disorder induced through high-energy ball milling, the authors successfully achieved a dense, highly conductive garnet electrolyte at just 500 °C, without the need for high-temperature sintering or sintering aids. This low-temperature pathway not only preserves lithium stoichiometry and avoids interfacial degradation but also enables strong interparticle connectivity critical for long-term cycling. The stable capacity and high Coulombic efficiency observed in the hybrid Li-metal cells confirm that a mechanochemically derived D-garnet can deliver electrochemical performance comparable to traditionally sintered counterparts while offering a scalable and energy-efficient processing route.

Kwon et al.’s work highlights the critical role of mechanochemical activation in enabling a sintering-free route for the fabrication of garnet-type solid electrolytes. By introducing structural disorder through high-energy ball milling, the authors effectively transform conventional crystalline precursors into an amorphous, deformable matrix, allowing for the formation of dense, interconnected microstructures at room temperature.

Beyond the benchmark studies mentioned above, several other mechanochemically assisted approaches have been explored for oxide-type SEs. For example, Oleszak et al. demonstrated that mechanical milling followed by moderate heat treatment (~750 °C) significantly improved the formation of cubic-phase LLZO, achieving up to 90% phase purity, which is substantially higher than that obtained through direct calcination [65]. In another study, Kozawa et al. developed a combined wet ball-milling and water-vapor heat treatment strategy for the NASICON-type LATP system, enabling crystallization at temperatures as low as 350 °C. This approach also controlled particle growth while preserving high Li^+^ conductivity in the resulting compacts [66].

These studies highlight the versatility of mechanochemical methods—not only in reducing synthesis temperatures and enhancing phase selectivity but also in enabling novel pathways for nanostructure design, densification, and performance optimization across various oxide SEs systems.

#### 4.1.4. Mechanochemical Synthesis of Sulfide-Based SEs

Sulfide-based SEs are a cornerstone in advancing ASSBs due to their high ionic conductivity, low processing temperatures, and favorable mechanical properties. Mechanochemical synthesis, particularly ball milling, has emerged as a scalable and solvent-free method to produce these materials due to their simplicity, solvent-free nature, and scalability. Traditional dry ball milling methods, using binary or ternary precursors such as Li_2_S, P_2_S_5_, and LiCl, have enabled the synthesis of electrolytes like β-Li_3_PS_4_ (LPS) and argyrodite-type Li_6_PS_5_Cl. These routes typically rely on high-energy mechanical activation to overcome kinetic barriers in solid-state reactions, followed by post-annealing to achieve desired crystallinity and conductivity [67,68]. This section highlights recent representative studies, covering both conventional and emerging mechanochemical strategies for the synthesis of sulfide-based SSEs.

**Figure 15:**
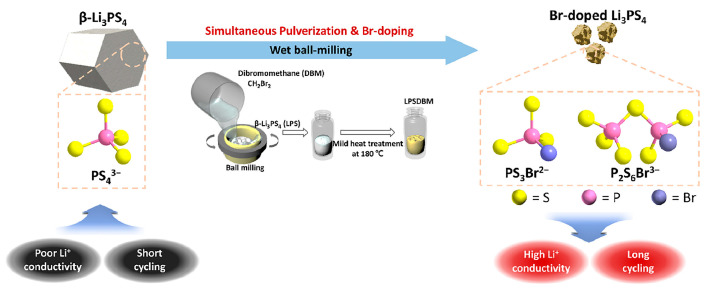
Preparation routes of LPSDBM. Reprinted with permission from Park, S. H. et al. [71], Energy Storage Materials, 2023, 63, 102985. Copyright © 2023 Elsevier B.V.

Elemental doping has become a widely adopted strategy for enhancing the Li-ion ionic conductivity of sulfide-based SEs [69,70]. Park et al. proposed an innovative mechanochemical wet doping strategy using dibromomethane (DBM), which serves as both a bromine source and an inert solvent medium [71]. As shown in Figure 15, pre-synthesized β-Li_3_PS_4_ is treated with DBM during high-energy milling, allowing for simultaneous Br substitution and particle size reduction. This strategy led to an eightfold increase in ionic conductivity, reaching 1.3 mS·cm^−1^ at only 1.14 at.% Br doping. Notably, the process maintained the original crystal structure of Li_3_PS_4_, indicating a non-destructive substitution mechanism. In contrast, the traditional doping methods usually involve mixing dopants (e.g., LiBr) with precursors in a one-step ball milling and heat treatment process [72], which often leads to issues such as low doping efficiency, formation of unwanted secondary phases, poor compositional uniformity, and inconsistent batch-to-batch performance. The successful application of wet ball milling for Br-doping in Li_3_PS_4_ underscores the transformative potential of mechanochemical strategies in the design and optimization of sulfide-based SEs. This approach effectively addresses the key limitations of conventional methods, offering improved dopant incorporation, structural integrity, and process control for the design and optimization of sulfide-based solid-state electrolytes.

Other attempts of developing sulfide SEs, such as Li_6_PS_5_Cl (argyrodite-type) and LGPS-type compounds, using mechanochemical-assisted synthesis further demonstrate the scope and potential of the mechanochemistry strategy. For instance, Hofer et al. demonstrated that by optimizing process parameters such as milling speed, media size, and filling ratio, β-Li_3_PS_4_ could be synthesized in under 5 h while achieving desirable ionic conductivity and microstructure control [34]. Similarly, Lu et al. reported a single-step high-energy ball milling approach for synthesizing glassy-ceramic Li_10_GeP_2_S_12_ with high ionic conductivity, emphasizing the feasibility of amorphization and crystallization control via milling time alone [73]. Beyond synthesis, mechanochemistry also facilitates compositional tuning and structural design. Kanazawa et al. reported the preparation of metastable hexagonal Li_4_SnS_4_, a Sn-based sulfide with improved air stability compared to conventional phosphorus-based SEs. This work highlights the versatility of mechanochemical techniques in accessing non-equilibrium phases that are difficult to obtain through traditional routes [74].

These studies demonstrate that mechanochemical synthesis is not merely a cost-effective and scalable processing technique but also a versatile platform for tailoring the structure, composition, and performance of sulfide-based SEs.

Table 2 compiles key information from representative studies discussed in the text, including electrolyte composition, synthesis method, ionic conductivity, and notable features.

### 4.2. Anode Material

In ASSBs, the anode functions as the primary host for Li insertion and extraction, playing a crucial role in determining the overall energy density, cycle life, electrochemical performance, and safety. The anode materials are expected to meet a range of stringent performance criteria to ensure energy efficiency, structural stability, and improved safety. These include high theoretical capacity, low density, and low electrochemical potential to maximize energy density, as well as chemical and mechanical stability for safe handling and reliable operation. On the other hand, the anode materials typically experience volume change during repeated charging and discharging, leading to issues such as volume-induced mechanical degradation, unstable interfaces, and dendritic growth which could cause disfunction or even safety concerns [75,76]. As such, controlling volume expansion has become a key design consideration in the development of next-generation anode materials for ASSBs.

**Figure 16:**
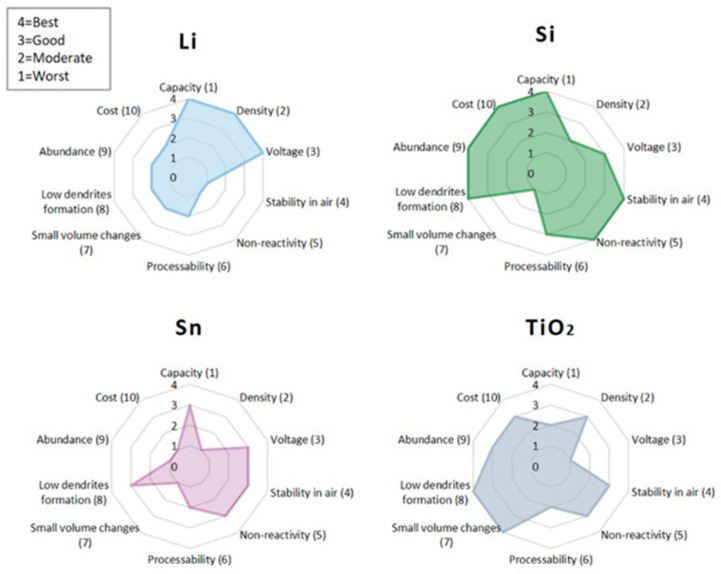
Radar chart comparing key properties of various anode materials used in solid-state batteries. Adapted with permission from Ref. Jetybayeva et al. [75]. ©2023 Elsevier.

Figure 16 provides a comparative assessment of typical anode materials used in ASSBs, including lithium (Li), silicon (Si), tin (Sn), and titanium dioxide (TiO_2_), based on a set of critical performance criteria. These criteria include capacity, density, voltage, stability in air, non-reactivity, processability, volume change, dendrite formation tendency, abundance, and cost. Each property is ranked on a scale from 1 (worst) to 4 (best). The radar chart visually highlights the trade-offs among different anodes: Li offers the highest capacity but suffers from poor air stability and high dendrite risk while TiO_2_ exhibits excellent safety and stability but with much lower capacity. Si demonstrates a promising balance of capacity and air stability, albeit with significant volume expansion concerns.

Traditional synthesis methods for anode materials in solid-state batteries vary widely depending on the material type, including slurry casting, hydrothermal synthesis, spray granulation, anodization, and atomic layer deposition, each offering specific advantages in controlling structure, morphology, and electrochemical performance. These techniques have been successfully applied to materials such as Si, Sn, and TiO_2_, enabling improved capacity, stability, and scalability across various solid-state battery configurations [77,78,79,80,81].

Among the candidates of anode materials, Si is promising due to its ultra-high theoretical capacity (~4200 mAh g^−1^) [82], natural abundance, and low operating potential. However, its practical application is hindered by severe volume expansion during cycling, which can lead to mechanical degradation and poor cycling stability. Additionally, conventional fabrication methods for silicon anodes often involve complex processing, high costs, and limited scalability [83,84]. Recently, mechanochemical synthesis has emerged as a simple, solvent-free approach that can simultaneously induce chemical bonding and reduce particle size, resulting in structures that are more tolerant to volume changes during cycling. For instance, Koraag et al. developed a two-step high-energy ball milling method to synthesize silicon/carbon nanotube (Si/CNT) composite anode [85]. In the first step, wet ball milling was used to reduce the silicon particle size and generate more rounded particles which is an advantageous morphology for minimizing stress during volume changes. In the second step, the nano-silicon was subjected to dry ball milling (1200 rpm, 2 h) with single-walled carbon nanotubes (CNTs). This process not only ensured intimate contact between Si and CNTs but also induced the formation of Si-C covalent bonds at the interfaces, as confirmed by XPS and Raman spectroscopy [85]. Moreover, the improved structural integrity and stable SEI formation achieved by the covalently bonded Si/CNT composite may contribute to the suppression of dendritic growth on the anode. As reported by Koraag et al., the CNT matrix helps to prevent the pulverization and detachment of silicon particles during cycling, while the formation of strong Si–C bonds maintains electrode integrity. These features, together with the enhanced Coulombic efficiency and reduced SEI build-up, suggest a more uniform lithium deposition behavior, which is beneficial for minimizing dendrite formation during repeated charge/discharge cycles.

**Figure 17:**
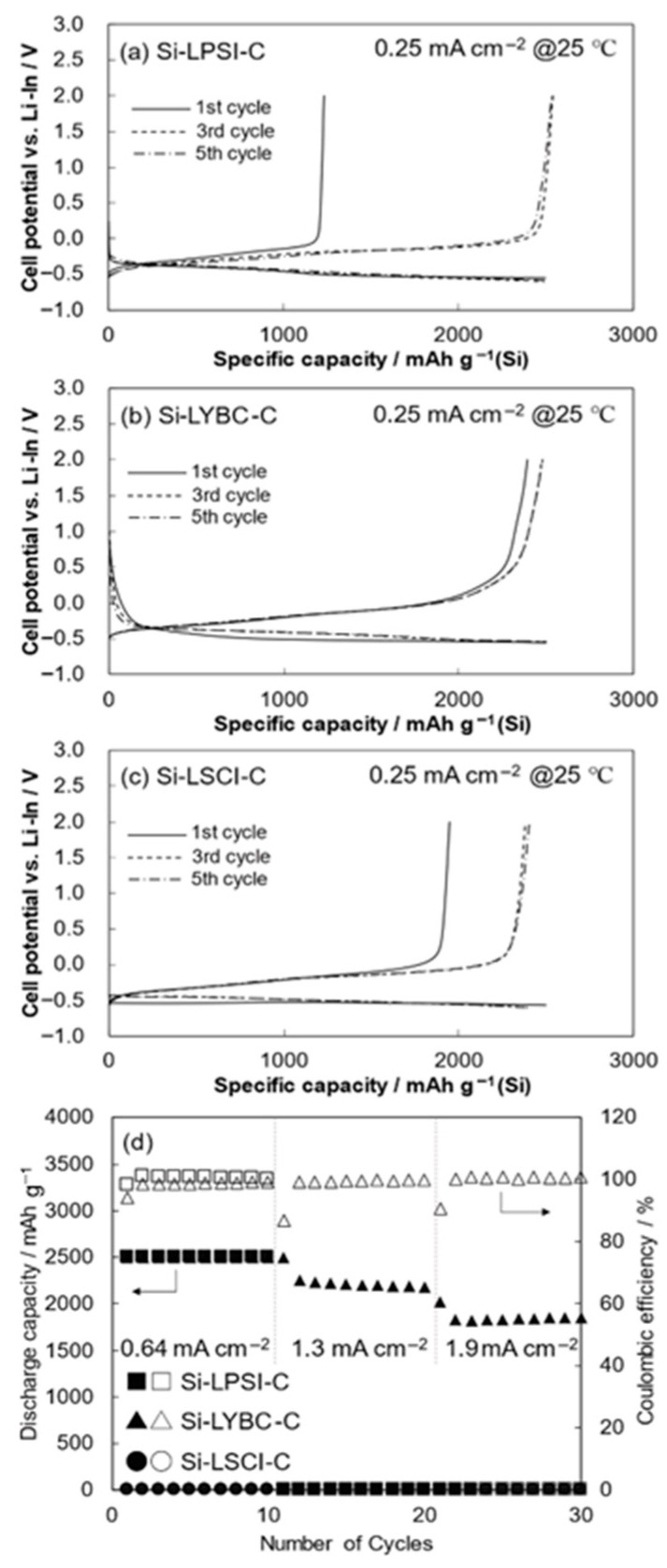
Half-cell charge and discharge curve. (**a**) Si-LPSI-C, (**b**) Si-LYBC-C, and (**c**) Si-LSCI-C. (**d**) Rate performances of each cell. Reproduced with permission from H. Nagata and K. Kataoka [86], J. Power Sources, 623, 235443 (2024). © 2024 Elsevier.

Beyond its role in synthesizing anode composites, ball milling can also serve as an effective diagnostic tool to assess interfacial compatibility between electrode materials and SEs. By mechanically accelerating potential chemical reactions at the solid–solid interface, this technique enables the early identification of undesirable side reactions, such as the formation of insulating interphases prior to full-cell assembly. For example, Nagata and Kataoka employed the ball milling method to investigate the interfacial reactivity between Si anode and three types of SEs: a sulfide-based LPSI, an oxide-based LSCI, and a halide-based LYBC [86]. In Figure 17, which is obtained from the study by Nagata et al., the half-cell electrochemical performance of Si-based composite anodes prepared via mechanochemical milling with various solid electrolytes (LPSI, LYBC, and LSCI) is presented. Despite all electrodes being fabricated under identical ball milling conditions, substantial differences in first-cycle delithiation capacity and rate performance were observed. Notably, the Si–LYBC–C electrode exhibited a high initial coulombic efficiency (~96%) and excellent rate capability, whereas Si–LPSI–C and Si–LSCI–C suffered from significant irreversible capacity losses and poor high-rate performance.

X-ray photoelectron spectroscopy (XPS) is a surface-sensitive analytical technique used to determine the elemental composition, chemical states, and electronic environment of elements within the top few nanometers of a material. By measuring the kinetic energy of electrons emitted from a material upon X-ray irradiation, XPS provides quantitative and chemical bonding information, making it particularly valuable for investigating interfacial reactions and surface modifications in battery materials. In Nagata et al.’s study, X-ray photoelectron spectroscopy (XPS) revealed that LPSI and LSCI formed insulating interphases (Si–S and SiOx, respectively) while LYBC remained chemically stable. These findings were supported by corresponding shifts in the P 2p and S 2p (for LPSI) and S 2p (for LSCI) spectra, indicating redox activity during ball milling. In contrast, the Si–LYBC composite maintained a predominantly elemental Si signal with no evidence of chemical bonding or reduction in the halide components (Cl, Br, Y), confirming its chemical inertness. These results highlight the critical role of interfacial reactions during mechanochemical processing and explain the superior electrochemical reversibility observed for the Si–LYBC–C electrode. As a result, LYBC–Si composites retained higher electronic conductivity and demonstrated superior electrochemical performance, including higher reversible capacity and higher initial Coulombic efficiency. In full-cell tests, the LYBC–Si configuration achieved an impressive energy density of 470 Wh kg^−1^ [86].

### 4.3. Cathode Material

Cathode active materials are another critical component in ASSBs, serving as the primary Li storage medium when the cell is charged. During discharging, Li ions transport from the anode to the cathode, resulting in a lithiated cathode in the discharged state. While in a charged cell, Li ions transport from cathode to anode, and the cathode material is delithiated [87].

To ensure safety and high cycling stability in ASSBs, cathode materials must meet several essential criteria. First of all, high loading of cathode active material with high energy density is critical to improve battery capacity [87]. Moreover, the ability to transfer Li ion within the cathode is also critical to avoid poor utilization of active materials. In addition, cathode materials also require excellent mechanical and electrochemical stability to avoid unwanted side reactions (e.g., interfacial decomposition) and mechanical cracking or detachment within the cathode material and at the cathode/electrolyte interface during the charging and discharging process. This is especially crucial for Ni-rich layered oxides (e.g., the Ni-rich NCM), which can shrink during cycling [88]. However, cathode materials for ASSBs continue to face a number of challenges, including poor interfacial contact, chemical instability, structural degradation due to volume changes, and process challenges during preparation. These issues collectively hinder their electrochemical performance and long-term stability.

**Figure 18:**
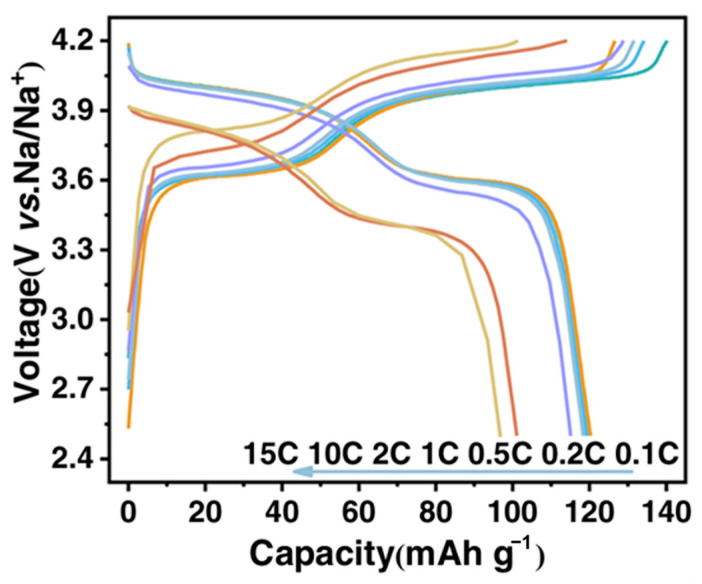
Charge–discharge profiles of NVOPF in Na half-cell at rates from 0.1 C to 15 C. Adapted with permission from Shen et al. [37], Nat. Commun. 12, 2848 (2021). © Springer Nature.

Recently, mechanochemical methods have been adopted as green and efficient synthesis strategy for preparing cathode materials. These methods not only simplify the process and reduce energy consumption but also facilitate nanoscale particle formation and homogeneous mixing of materials, enhancing the electrochemical activity and interfacial properties of cathode materials. Therefore, they hold significant promise for the scalable preparation of high-performance cathode materials. In one example, Mohamed et al. successfully synthesized phase-pure Li_2_FeSO via a solvent-free mechanochemical route and systematically characterized its structural and thermal stability [89]. Extending this approach beyond the Li-ion system, Shen et al. employed high-energy ball milling to rapidly synthesize phase-pure Na_3_(VOPO_4_)_2_F (NVOPF) for sodium-ion batteries [37]. Figure 18 presents the rate capability of the as-prepared NVOPF electrode measured in half-cell configuration against sodium metal, the different colored curves represent the charge–discharge voltage profiles of the NVOPF electrode at various C-rates (from 0.1C to 15C), illustrating its rate capability. The charge–discharge profiles were recorded at gradually increasing current densities ranging from 0.1 C to 15 C within a voltage window of 2.5–4.2 V (vs. Na/Na^+^). Two distinct voltage plateaus around 4.10/4.05 V and 3.65/3.60 V have been observed, indicative of reversible sodium intercalation/deintercalation. The electrode delivers a discharge capacity of 120.7 mAh g^−1^ at 0.1 C, retaining 96.5 mAh g^−1^ even at a high rate of 15 C, demonstrating excellent rate performance. The increasing voltage hysteresis at higher rates is attributed to the intrinsic low electronic conductivity of polyanionic compounds.

Through in situ co-milling with Ketjen Black, a nanocomposite cathode was obtained that delivered an impressive reversible capacity of 142.2 mAh g^−1^, excellent rate capability (113 mAh g^−1^ at 20 C), and remarkable cycling stability, retaining 98% of its capacity over 10,000 cycles [37]. These studies highlight the broad applicability and scalability of mechanochemical synthesis for advanced cathode material development across multiple battery chemistries.

## 5. Mechanochemically Induced Polymerization and Its Application in Energy Storage Materials

In addition to inorganic systems, mechanochemical methods have recently demonstrated significant potential in the synthesis of organic materials, including polymer. Solid-phase synthesis of polymers can be realized by applying mechanical energy through ball milling and other means, which can effectively initiate the polymerization reaction [90,91]. This section focusses on the underlying mechanisms of mechanochemically induced polymerization and its emerging applications in energy storage materials.

### 5.1. Mechanochemically Induced Polymerization

Depending on the polymer structure, current research on mechanically induced polymerization primarily focuses on two categories: linear polymers and porous polymers [92]. For linear polymers, mechanochemical methods can induce polymerization through different mechanisms, with free radical polymerization and ring opening polymerization (ROP) being the mostly widely explored. In free radical polymerization, free radicals can be generated during ball milling through the incorporation of radical initiators, such as azobisisobutyronitrile (AIBN) or benzoyl peroxide (BPO), which in turn initiates the polymerization of monomers such as styrenes, acrylics, methacrylics, and so on. Notably, recent studies have demonstrated the feasibility of initiator-free radical polymerization under mechanochemical conditions. In such cases, mechanical energy alone can drive radical generation, offering a more sustainable and environmentally friendly approach aligned with green chemistry principles. One proposed mechanism involves the generation of free radicals when the collision energy between milling balls and jars exceeds the work function of the milling materials, enabling electron transfer to the monomer molecules [93,94].

**Figure 19:**
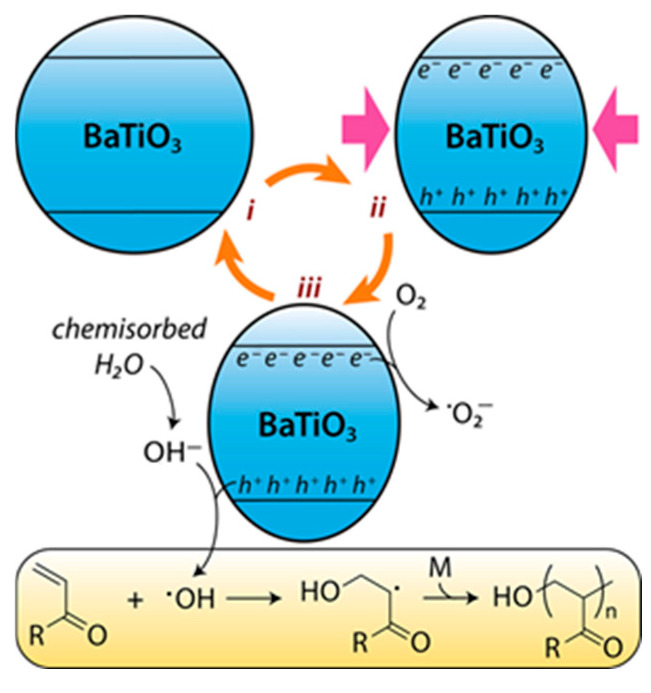
Radical generating mechanism. Reprinted with permission from Nothling et al. [91], Angew. Chem. Int. Ed., 2023, 62, e202214061. © Wiley-VCH.

Another interesting method utilizes the local electric field generated by mechanical stress during ball milling of piezoelectric materials such as Barium Titanate (BaTiO_3_). This phenomenon, known as piezocatalysis, induces the electron transfer reaction between initiators and monomers through charge migration, generating free radicals, and initiating polymerization. For example, Nothling et al. demonstrated solid-state free radical polymerization of acrylate monomers via this piezocatalytic mechanism [91]. In this mechanism, piezoelectric materials such as BaTiO_3_ under mechanical stress produce charge separation, generating surface regions enriched with electrons and holes. These electrons and holes can drive redox reactions by oxidizing chemisorbed water to generate hydroxyl radicals or by reducing oxygen molecules to form superoxide radicals. All these reactive radicals subsequently initiate the free radical polymerization of monomers such as acrylates to form polymer chains [91]. The radical generation pathway involved in this process is shown in Figure 19.

Mechanochemical methods also offer significant synthetic advantages in the preparation of porous polymers. Porous polymers, such as covalent organic frameworks (COFs) [95] and highly cross-linked porous polymers (HCPs), often suffer from poor monomer diffusivity, undesirable side reactions, and low yields when prepared using traditional solution systems. Mechanochemical synthesis, on the other hand, can enable rapid polymerization through direct physical mixing and mechanical activation, facilitating efficient condensation or cross-linking reactions and yielding structurally robust polymer networks with tunable porosity within minutes. For example, Schiff base-type COFs can be synthesized rapidly by ball milling of monomers containing aldehyde and amine groups, without the need for catalysts or solvents [96]. Moreover, liquid-assisted grinding (LAG) has been investigated to enhance reaction efficiency and improve the crystallinity of the resulting frameworks. These porous polymers have found widespread applications in areas such as gas absorption, heterogeneous catalysis, and as functional scaffolds in battery electrodes.

### 5.2. Mechanochemically Synthesized Polymer Materials for Energy Storage Applications

**Figure 20:**
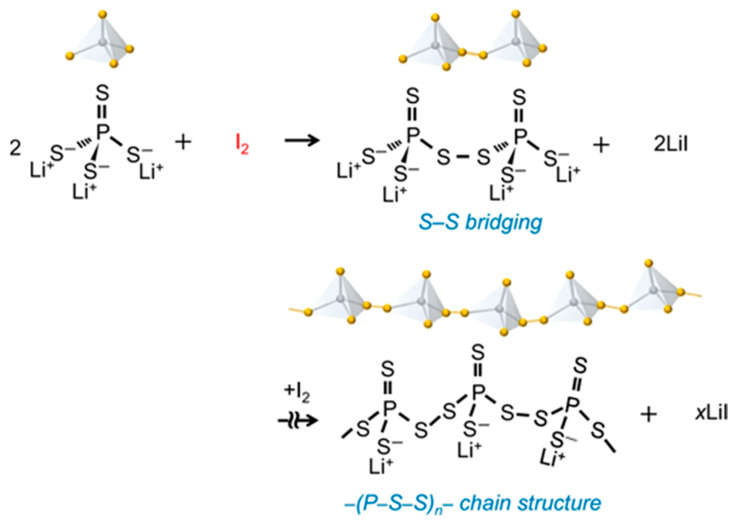
Diagram depicting I_2_-driven polymer formation from Li_3_PS_4_. Reproduced from Kato et al. [97] with permission. © 2021 Springer Nature.

Mechanochemically induced polymerization has emerged as a powerful strategy for synthesizing energy storage materials for applications in solid state batteries. By coupling mechanical force with reactive precursors, this solvent-free or low-solvent technique enables the formation of polymers with tailored ionic conductivity and mechanical resilience. A notable example is the development of Li-ion-conductive sulfide polymer binders through iodine-induced polymerization of Li_3_PS_4_, as demonstrated by Kato et al. [97]. As shown in Figure 20, the mechanochemical method, involving the direct ball milling of Li_3_PS_4_ with I_2_, facilitates the formation of structurally diverse polymer networks, potentially including branched and cyclic architectures. The reaction involves the coupling of PS_4_^3−^ anions through oxidative linkage by I_2_, forming disulfide-bridged dimers and eventually leading to the generation of an elastic polymer framework composed of (–P–S–S–)_n_ segments. The byproduct, formed by the combination of I- with Li^+^, also contributes to Li-ion conduction [97]. In their study, both mechanochemical and liquid-phase synthesis (LS) routes were evaluated to investigate structural robustness, tunability of material properties, and compatibility with various processing strategies. The polymer materials obtained through the mechanochemical method demonstrated significantly higher Li-ion conductivity of 2.9 × 10^−4^ S cm^−1^ for a Li_3_PS_4_ − I_2_ (1:1) molar ratio, which can be attributed to enhanced crosslinking density and more effective incorporation of disulfide linkages. In contrast, the LS route produces more uniform chain-like polymers with superior solubility and processability, forming stable gels in anisole. However, the resulting Li-ion conductivity is significantly lower (~2.3 × 10^−6^ S cm^−1^), even after thermal treatment [97].

**Figure 21:**
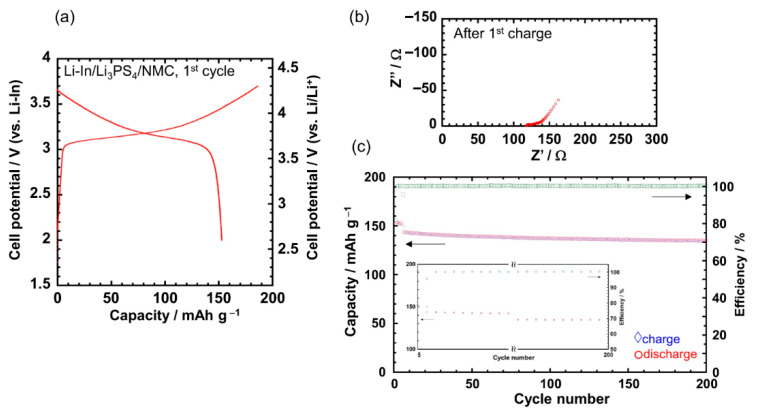
Electrochemical performance of an all-solid-state Li–In/Li_3_PS_4_/NMC cell containing Li_3_PS_4_–I_2_ (1:1) as a polymer binder. (**a**) First charge–discharge profile at 30 °C, (**b**) Nyquist plot after the first charge, and (**c**) cycling performance up to 200 cycles. Reproduced from Kato et al. [97] with permission. © 2021 Springer Nature.

The electrochemical performance of the Li_3_PS_4_-I_2_ (1:1) polymer synthesized via mechanochemical ball milling was further validated through all-solid-state cell testing, as shown in Figure 21 [97]. When used as a Li^+^-conductive binder in a Li-In/Li_3_PS_4_/NMC cell, the material exhibited excellent cycling stability, with a capacity retention of 93.8% after 200 cycles (Figure 21). The initial charge–discharge curves (Figure 21a) displayed typical behavior of the NMC active material within a voltage range of 2.6–4.3 V vs. Li/Li^+^, with no apparent side reactions, indicating electrochemical compatibility. Moreover, the Nyquist plot obtained after the first charge (Figure 21b) revealed a small cell resistance, suggesting that the sulfide polymer binder introduced minimal additional impedance. These results demonstrate that the ball milled Li_3_PS_4_-I_2_ polymer not only enables flexible electrode fabrication but also contributes to long-term electrochemical stability in ASSBs.

These findings highlight the trade-offs between ionic performance and processability, suggesting that while liquid-phase synthesis is advantageous for scalable, solution-based electrode fabrication, mechanochemical synthesis routes offer superior electrochemical performance.

**Figure 22:**
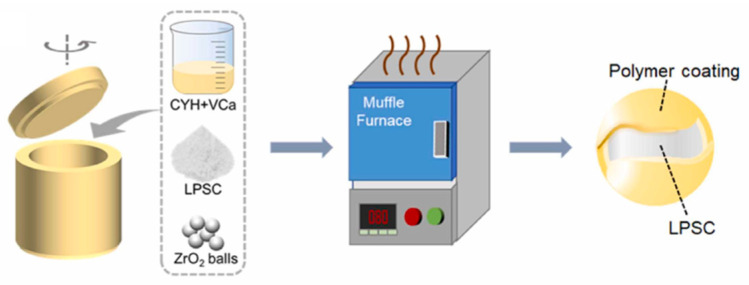
Process route for synthesizing polymerization-coated sulfide composite solid electrolyte. Adapted with permission from Yuan et al. [98], Copyright © 2024 Elsevier.

Building upon these developments, more recent studies have further explored the chemical reactivity enabled by mechanochemical processing environments. For example, Yuan et al. [98] introduced a wet ball milling–assisted in situ polymerization strategy to prepare ultra-soft polymer-coated sulfide composite electrolytes, as shown in Figure 22 [98]. In this work, vinylene carbonate (VCa) monomers were mixed with Li_5_._5_PS_4_._5_Cl_1_._5_ (LPSC) and a radical initiator (AIBN) in cyclohexane solvent. The mechanical energy provided by ball milling simultaneously facilitated particle refinement and initiated the polymerization of VCa, leading to the uniform deposition of poly(vinylene carbonate) (PVCa) on the surface of LPSC particles. This polymer coating served multiple critical functions, including providing mechanical flexibility, improved particle–particle contact, and acted as an electron-blocking layer for suppressing Li dendrite growth.

The composite electrolyte demonstrated excellent electrochemical performance, including dendrite-free Li plating/stripping for over 2000 h, a high critical current density of 2.0 mA cm^−2^, and excellent cycling performance when paired with high-loading NCM cathodes. Unlike traditional dry milling routes, this wet mechanochemical strategy highlights the potential of mechanochemical environments, not only for structural refinement but also for inducing targeted chemical transformations, such as polymerization and engineering functional interfaces.

**Figure 23:**
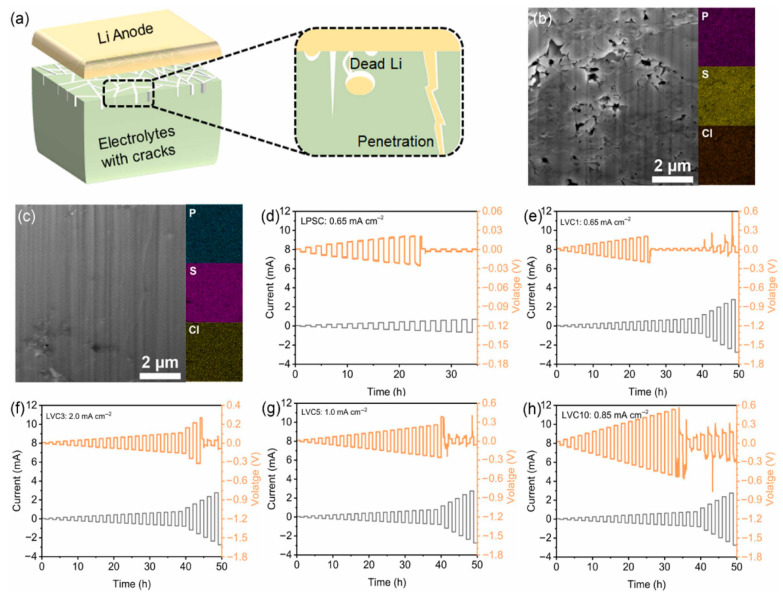
(**a**) Schematic picture of lithium dendrite with cracks. (**b**) SEM image and EDS mapping results of (**b**) LSPC and (**c**) LVC3 (**d**–**h**) critical current density results. Reprinted with permission from Yuan et al. [98], Copyright © 2024 Elsevier.

Figure 23 from Yuan et al. offers a compelling comparison between conventionally processed LPSC and the mechanochemically synthesized, polymer-coated LVC3 composite electrolyte. The cold-pressed LPSC sample (Figure 23b) reveals a loosely packed structure with visible interparticle voids and cracks which act as favorable sites for lithium dendrite growth, leading to premature short-circuiting. In contrast, the LVC3 sample (Figure 23c), fabricated via wet ball milling followed by in situ polymerization, forms a much denser and more cohesive microstructure, effectively eliminating such defects. These morphological differences are clearly visualized by FIB-SEM and further confirmed by energy-dispersive X-ray spectroscopy (EDS) mapping, which shows uniform distribution of key elements (S, P, C), indicating successful and homogeneous polymer coating. This dense architecture not only enhances interfacial contact but also enables superior electrochemical stability, as evidenced by the substantial increase in critical current density (CCD). The CCD of LPSC is limited to only 0.65 mA cm^−2^, whereas LVC3 achieves a markedly higher value of 2.0 mA cm^−2^, attributed to the polymer layer’s ability to suppress electron leakage and accommodate mechanical stress. This improvement underscores the critical role of mechanochemical processing in achieving defect-free electrolyte structures and dendrite-resistant interfaces for solid-state lithium metal batteries.

**Figure 24:**
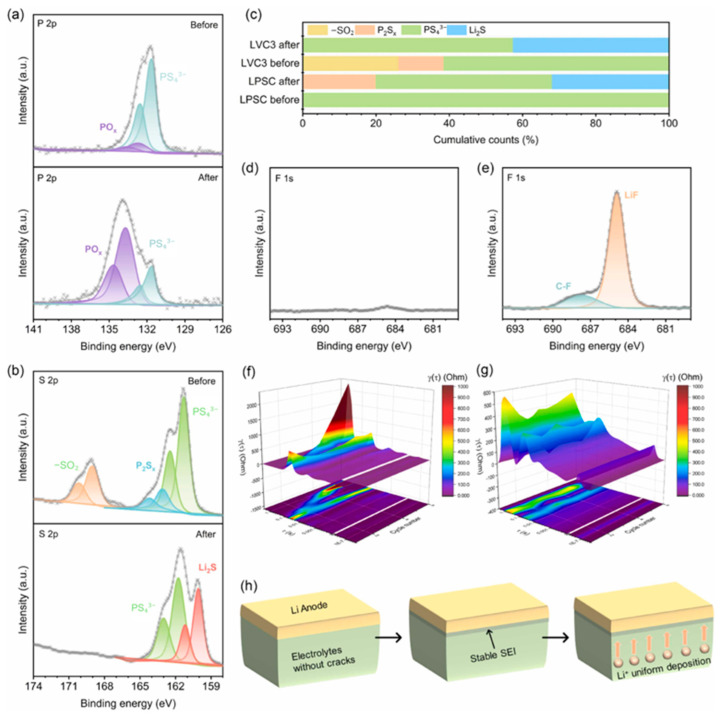
(**a**,**b**) XPS spectra of P 2p and S 2p at the LVC3–Li interface, showing partial decomposition. (**c**) Comparison of interfacial composition between LPSC and LVC3. (**d**,**e**) F 1s spectra indicating LiF formation in LVC3. (**f**,**g**) DRT impedance maps showing stable interfacial evolution in LVC3 versus LPSC. (**h**) Schematic of the interfacial stabilization mechanism by the LVC3 coating. Reprinted with permission from Yuan et al. [98], Copyright © 2024 Elsevier.

Yuan et al. further elucidated the degradation pathways of sulfide electrolytes in contact with lithium metal, comparing traditional LPSC and the polymer-coated LVC3. In the case of LPSC, the interface undergoes severe chemical degradation during cycling, leading to the formation of electrochemically inactive products such as Li_3_PS_4_, PO_x_, and P_2_S_x_, as confirmed by XPS analysis (Figure 24a–c). These byproducts result in rapid impedance growth and promote dendritic lithium penetration through the formation of porous and fractured interfacial layers (Figure 23b). In contrast, the polymer-coated LVC3 shows a more controlled degradation pathway. The coating decomposes partially into beneficial SEI components like lithium fluoride (LiF) and lithium phosphate, which help maintain interfacial stability and suppress dendrite formation. Remarkably, the proportion of intact PS_4_^3−^ units in LVC3 remains largely preserved (from 61.3% to 57.4%), indicating minimal structural breakdown. Distribution of Relaxation Times (DRT) analysis also reveals that LVC3 exhibits much slower and more stable impedance evolution than LPSC (Figure 24f–g), further confirming the effectiveness of the coating in mitigating chemomechanical degradation at the electrode–electrolyte interface.

Together, these studies illustrate how mechanochemical strategies are evolving from simple solid-state mixing techniques into versatile platforms for initiating chemical transformations, such as polymerization, capable of directing interfacial chemistry and enabling the design of high-performance materials for solid-state battery applications.

## 6. Conclusions and Outlook

Mechanochemistry has emerged as a powerful and versatile approach for the synthesis and engineering of solid-state battery materials. Originally employed as a simple solid-state mixing technique, it has evolved into a reaction platform capable of driving phase transformations, promoting chemical bonding, and initiating in situ polymerization. Representative studies, such as the synthesis of highly conductive sulfide electrolytes by ball milling by Kato et al. [97] and the construction of flexible composite electrolytes by in situ polymerization of monomers induced by ball milling by Yuan et al. [98], not only demonstrate the diverse functions of mechanochemistry in material construction but also prove its application prospects in improving interfacial contacts and electrochemical performance, inhibiting lithium dendrites, and other key challenges. These studies mark the evolution of mechanochemistry from a single physical processing tool to a strategic synthetic tool with the attributes of a “reaction platform”. In the past, mechanochemistry was mainly used for physical mixing, size reduction, or crystalline phase homogenization, and its role was limited to morphology control and preliminary mixing of materials. However, with the deepening of research, it has been gradually recognized that mechanical energy can not only provide external power but also stimulate substantial chemical reactions between reactants, such as crystal transformation, bond breaking and reorganization, and even induced polymerization and other complex processes. This paradigm shift from “physical processing” to “reaction-driven” enables mechanochemistry not only to synthesize highly reactive materials under extremely mild conditions but also to precisely regulate the microstructure and interfaces of the materials, giving them higher chemical functionality and engineering practical value. Therefore, mechanochemistry is gradually becoming an important platform technology that can stimulate new reaction pathways and construct multi-scale functional materials.

Looking ahead, the research of mechanochemical methods in the field of battery materials still has a broad space for development. On the one hand, with the advancement of equipment technology, the introduction of emerging means such as resonance acoustic mixing (RAM) and twin-screw extrusion (TSE) technology has provided mechanochemical synthesis with stronger continuity and mild processing capability, which is expected to promote it from the laboratory to large-scale production. On the other hand, the synergy between mechanochemistry and other energy input modes, such as mechanical-thermal, mechanical-electric field, mechanical-light, and other composite reaction strategies, is becoming an important direction to improve the reaction selectivity and modulation ability. In addition, with the help of functional construction strategies such as mechanical energy-induced polymerization, it is possible to introduce more complex chemical structures within or at the interface of materials to enhance the overall electrochemical performance. Future research should focus on gaining a deeper mechanistic understanding of reaction pathways and phase evolution during mechanochemical processing, as well as developing in situ characterization techniques to monitor these transformations in real time. Moreover, optimizing process parameters for scalability, improving batch-to-batch consistency, and tailoring the microstructure and interfacial properties of the resulting materials are essential for practical application. The integration of mechanochemical methods with other techniques—such as thermal, electrochemical, or photochemical activation—also holds great promise for designing advanced solid-state electrolytes and electrodes.

## Figures and Tables

**Table 1:** Overview of features from different ball milling techniques. Data summarized and adapted from Ref. [11].

Ball Mill Type	Ball Movement Mechanism	Cooling Feasibility	Scalability	Acceleration Level	Collision Frequency	Stressing Energy
Tumbling Mill	Drum rotation	✖ Very limited	★★★★☆	~1× *g*	Low	★★★☆☆
Planetary Mill	Rotation in centrifugal field	✖ Very poor	★☆☆☆☆	<150× *g*	Moderate	★★★★☆
Vibratory Mill	High-frequency vibration	△ Moderate	★★☆☆☆	<30× *g*	High	★★★☆☆
Agitator Mill	Rotating stirrer in fixed vessel	✔ Excellent	★★★★☆	Several 100× *g*	Very High	★★★☆☆

**Table 2:** Summary of lithium-ion conducting solid electrolytes synthesized by mechanochemical methods. The table includes information on electrolyte composition, synthesis route, ionic conductivity, testing temperature, key features, and reference work. RT denotes room temperature.

No.	Type	Electrolyte Composition	Synthesis Method	Li+ Conductivity (S/cm)	Testing Temp. (°C)	Key Features/Notes	Ref.
1	Halide type	Li_3_ErCl_6_	Ampoule synthesis	1.7 × 10^−5^	RT	High Er site disorder enhances ionic conductivity	[55]
			Ball milled	3.1 × 10^−4^	RT		
2	Halide type	Li_2_ZrCl_6_	Ball milled and annealed	4.46 × 10^−4^	RT	Mild heat treatment (100 °C) can largely enhance ionic conductivity	[51]
3	Halide type	Li_2_._25_Zr_0_._75_Fe_0_._25_Cl_6_	Ball milled and heat-treated	1 × 10^−3^	35 °C	The heterovalent ion substitution of Li_2_ZrCl_6_ with Fe^3+^ enhances Li^+^ conductivity	[56]
4	Halide type	Li_3_YCl_6_	Ball milled	4.9 × 10^−4^	25 °C	Stacking faults enhance lithium-ion conduction in Li_3_YCl_6_ by creating favorable pathways	[57]
			Solid-state synthesized	6.7 × 10^−5^	25 °C		
5	Oxide garnet type	Li_6_._5_La_3_Zr_1_._5_Ta_0_._5_O_12_ (LLZTO)	Ball milled, pelletized, and heat-treated	3.5 × 10^−4^	25 °C	Sintering-free, cubic phase at 500 °C, high density	[64]
6	Oxide NASICON type	Li_1_._3_Al_0_._3_Ti_1_._7_(PO_4_)_3_ (LATP)	Wet ball milled and heat-treated in water vapor	3.5 × 10^−4^	25 °C	Crystallization from amorphous achieved at 350 °C in water vapor	[66]
7	Sulfide-Based type	Li_6_PS_5_Cl (LPSC)	Ball milled	3.5 × 10^−4^	20 °C	Synthesis route affects phase purity, densification, and Li^+^ transport; Ampcera yields optimal microstructure	[67]
			Ball milled and annealed	6.0 × 10^−4^	20 °C		
			Li_6_PS_5_Cl purchased from Ampcera	1.1 × 10^−3^	20 °C		
			Li_6_PS_5_Cl purchased from NEI	9.6 × 10^−4^	20 °C		
8	Sulfide-Based type	Br-doped Li_3_PS_4_ (LPS)	Ball milled with DBM	1.3 × 10^−3^	30 °C	Use of (DBM) as Br-source and solvent in a ball-milling process	[71]
9	Sulfide-Based type	0.98Li_6_PS_5_Cl–0.02YCl_3_	Ball milled	1.3 × 10^−2^	50 °C	Multivalent cation doping greatly boosted ionic conductivity above 50 °C	[72]
10	Sulfide-Based type	Li_3_PS_4_ (LPS)	Ball milled	3 × 10^−4^	RT	Maximum grinding efficiency with lowest specific energy consumption was achieved using large media, high speed, and moderate filling ratio	[34]
11	Phospho sulfide	Li_10_GeP_2_S_12_	Ball milled	1.07 × 10^−3^	RT	A one-step method for synthesizing glassy-ceramic Li_10_GeP_2_S_12_ using high-energy ball milling was developed	[73]
			Heat-treated after ball milling	3.27 × 10^−3^	RT		
12	Sulfide-Based type	Li_4_SnS_4_	Heat-treated after ball milling	1.1 × 10^−4^	RT	Crystalline Li_4_SnS_4_ with orthorhombic symmetry was obtained through mechanochemical synthesis and post-annealing	[74]
